# Care pathways of sepsis survivors: sequelae, mortality and use of healthcare services in France, 2015–2018

**DOI:** 10.1186/s13054-023-04726-w

**Published:** 2023-11-10

**Authors:** Fanny Pandolfi, Christian Brun-Buisson, Didier Guillemot, Laurence Watier

**Affiliations:** 1 Epidemiology and Modeling of Bacterial Evasion to Antibacterials Unit (EMEA), Institut Pasteur, Université Paris Cité,, Paris, France; 2 Centre de recherche en Epidémiologie et Santé des Populations (CESP), Institut National de la Santé et de la Recherche Médicale (INSERM), Université de Versailles Saint Quentin-en-Yvelines/Université Paris Saclay, Paris, France; 3 AP-HP, Paris Saclay, Public Health, Medical Information, Clinical Research, Le Kremlin-Bicêtre, France

**Keywords:** Acute care, Healthcare pathway, Infection, Intensive care unit, Medical administrative database, Post-discharge, Rehabilitation, Sepsis

## Abstract

**Background:**

Individuals who survive sepsis are at high risk of chronic sequelae, resulting in significant health-economic costs. Several studies have focused on aspects of healthcare pathways of sepsis survivors but comprehensive, longitudinal overview of their pathways of care are scarce. The aim of this retrospective, longitudinal cohort study is to identify sepsis survivor profiles based on their healthcare pathways and describe their healthcare consumption and costs over the 3 years following their index hospitalization.

**Methods:**

The data were extracted from the French National Hospital Discharge Database. The study population included all patients above 15 years old, with bacterial sepsis, who survived an incident hospitalization in an acute care facility in 2015. To identify survivor profiles, state sequence and clustering analyses were conducted over the year following the index hospitalization. For each profile, patient characteristics and their index hospital stay and sequelae were described, as well as use of care and its associated monetary costs, both pre- and post-sepsis.

**Results:**

New medical (79.2%), psychological (26.9%) and cognitive (18.5%) impairments were identified post-sepsis, and 65.3% of survivors were rehospitalized in acute care. Cumulative mortality reached 36.6% by 3 years post-sepsis. The total medical cost increased by 856 million € in the year post-sepsis. Five patient clusters were identified: home (65.6% of patients), early death (12.9%), late death (6.8%), short-term rehabilitation (11.3%) and long-term rehabilitation (3.3%). Survivors with early and late death clusters had high rates of cancer and primary bacteremia and experienced more hospital-at-home care post-sepsis. Survivors in short- or long-term rehabilitation clusters were older, with higher percentage of septic shock than those coming back home, and had high rates of multiple site infections and higher rates of new psychological and cognitive impairment.

**Conclusions:**

Over three years post-sepsis, different profiles of sepsis survivors were identified with different mortality rates, sequels and healthcare services usage and cost. This study confirmed the importance of sepsis burden and suggests that strategies of post-discharge care, in accordance with patient profile, should be further tested in order to reduce sepsis burden.

**Supplementary Information:**

The online version contains supplementary material available at 10.1186/s13054-023-04726-w.

## Background

Sepsis is a potentially life-threatening syndrome of systemic organ dysfunction caused by a dysregulated response to infection and a leading cause of global morbidity and mortality, responsible for several millions of deaths worldwide [1, 2]. Moreover, sepsis survivors face increased risks of physiological impairment, neurological disorders, psychological trauma and death [3]. In response to this major public health problem, at the World Health Assembly in 2017, the World Health Organization urged member states to improve epidemiological knowledge, prevention, diagnosis and management of sepsis [1, 2].

Medical administrative databases are widely used to characterize and quantify the burden of sepsis, and methodologies have been developed to maximize the sensitivity and specificity of case ascertainment using the standardized international definition of sepsis [4–8]. Using such methods, we previously estimated 403 cases of sepsis per 100,000 inhabitants in France in 2019 and a 90-day mortality rate of 31% [4]. This study highlighted the high burden of sepsis in France, with a short-term mortality consistent with previous publications [9–11]. However, compared to such short-term outcomes, descriptions of longer-term patient care pathways and estimates of post-sepsis morbidity, mortality and costs of care among sepsis survivors following their initial episode of sepsis are relatively scarce [3].

Characterizing long-term outcomes and use of health services among sepsis survivors is challenging due to the diversity of patient profiles and because healthcare pathways are complex dynamic processes [12] Several studies have focused on particular aspects of healthcare pathways of sepsis survivors, including hospital readmission, encounters with healthcare systems before or after sepsis and the incidence of morbidity post-sepsis [3, 9, 11, 13–17]. However, these approaches have failed to describe the overall profile of all sepsis survivors while providing a comprehensive, longitudinal overview of their pathways of care. Moreover, assessment of health impairment and healthcare use over a period longer than 1-year post-sepsis are scarce [3, 17, 18].

Initially used in social science, state sequence analysis (SSA) was more recently applied to the study of health care pathways [19–23]. This method can describe the temporal dimension of healthcare consumption and allows the identification of different care patterns and patient profiles. We applied state sequence analysis to exhaustive patient data from French medical administrative databases to identify various sepsis survivor profiles (“clusters”) based on the differences between their healthcare pathways in the year following their index hospitalization. In this retrospective, longitudinal cohort study, we also provide a comprehensive overview of the healthcare use and costs, sequels and mortality of sepsis survivors hospitalized with bacterial sepsis in France according to patient profiles over the 3 years following their index hospitalization.

## Methods

### Data sources, definitions and study population

This study consisted of a secondary data analysis of a national cohort of patients with bacterial infections admitted to hospitals in France. Therefore, only cases of sepsis of presumed bacterial etiology (henceforth referred to simply as *sepsis*) were included. The French national healthcare database (Système National des Données de Santé: SNDS) was used for the analysis (See Additional file 1: Methods). Sepsis was identified in the PMSI database as a combination of explicit sepsis and implicit sepsis [2, 6, 8, 24] (See Additional file 1: Methods and Table S1). More details about this selection can be found in our previous study [5].

The study population included sepsis survivors above 15 years old with an index hospital stay for sepsis ending in 2015. In order to select index sepsis-related hospitalizations only, stays with a sepsis-related hospitalization within the previous 12 months were excluded. Only index hospital stays in an acute care facility (Medicine, Surgery and Obstetrics: MSO) were considered. Stays shorter than one day and not ending in patient death were excluded. The data covered the period 1-year prior to the index hospitalization and the 3 subsequent years.

### State sequence analysis (SSA) of sepsis care pathways

State sequence analysis is a methodology used to describe longitudinal trajectories of individuals through the analysis of sequential categorical data (sets of sequences) [25].

In this study, a 365-day sequence was built for each sepsis survivor beginning at the end of their index hospitalization, thus representing their 1-year post-discharge care pathway. The sequence was divided into weekly units to obtain a good balance between representativeness and calculation power, leading to 52 units for each sequence. For each unit, seven states were considered: death of the patient (DEAD); inpatient hospitalization in an acute care facility (MSO_full), a rehabilitation facility (REHAB_full) or a psychiatric facility (PSY_full); hospital-at-home (HAH); day care in an acute care, rehabilitation or psychiatric facility (DAYCARE); and staying at home with or without ambulatory care (HOME). A SSA was conducted, and the sequences were grouped in different clusters using two complementary clustering methods: Ascendant Hierarchical Clustering based on Ward method (AHC) and Partitioning Around Medoids (PAM) (See Additional file 1: Methods). The sequence was built with SAS Enterprise Guide 7.1 (7.100.5.6214). R was used to compute the distance between sequences and cluster the sequences using TraMineR, Stats and WeightedCluster packages.

### Descriptive statistics

#### Patient characteristics, index hospital stay and healthcare use

Patient and incident sepsis stay characteristics were described for each cluster and for all survivors. This includes sex, age, Charlson index and detailed comorbidities, hospital discharge, length of stay, septic shock, ICU admission and infection site (See Additional file 1: Table S2). As the data cover the national population and due to the questionable significance of p-value for very large samples, statistical tests were not conducted to compare clusters [26, 27].

To assess the variations in the burden of care pre- and post-sepsis, healthcare use and monetary costs were calculated for all sepsis survivors over the 12 months before and 36 months after their index hospitalization and for each group of survivors (clusters) that were generated by the SSA and clustering analysis. Healthcare use included all inpatient hospitalization, day care in acute care, rehabilitation or psychiatry, hospital-at-home, outpatient hospital visits and ambulatory care received in the community (visits to general practitioners, specialists and nursing and physiotherapy care (including speech therapists)). To facilitate comparison between clusters by accounting for potential differences in survival, two additional outcomes were calculated: the hospitalization ratio (number of days occupied by hospitalization/number of days when the patient is alive) and the ambulatory visits ratio (number of days occupied by ambulatory visits/number of days when the patient is neither dead nor hospitalized) (See Additional file 1: Methods). The number of patients who died after their index hospitalization, the number who had prevalent morbidities and the number who acquired post-sepsis sequelae were also calculated, as were the amount of time spent alive and at home during the year following the incident hospitalization. Additionally, the total and median per-patient costs, pre- and post-sepsis, were calculated (median, interquartile range) for the index sepsis-related hospitalization as well as for the ambulatory care and hospital care (inpatient and day care). While less adapted to skewed distribution, but used in some publications, mean and 95% CI were also calculated. The difference between these pre- and post-sepsis costs was calculated to estimate excess healthcare cost following the incident sepsis episode. All annual outcomes were calculated for each of the following 2 years (2017 and 2018) for the patients who survived the first-year post-sepsis (2016).

#### Cognitive, psychological and medical impairment post-sepsis

Cognitive, psychological and medical impairment were assessed for all survivors and for each group of survivors (clusters). The prevalence of post-sepsis morbidities in the 12 months following the index sepsis episode was identified based on ICD-10 codes and CCAM codes (Common Classification of Medical Acts) and grouped into three domains (cognitive, psychological or medical) using a methodology adapted from Fleischmann et al*.* [3] (See Additional file 1: Methods). Morbidities recorded during the 12 months preceding the index hospitalization were considered as prevalent, and those subsequent to the index hospitalization as incident post-sepsis sequelae. Finally, the prevalence and incidence of dialysis and long-term mechanical ventilation were calculated for all survivors, each group of survivors and for those belonging to populations at particularly high risk (respectively, patients with chronic renal disease without dialysis and patients with chronic pulmonary disease without long-term mechanical ventilation) (See Additional file 1: Methods).

## Results

### Patient characteristics, sequels and healthcare use and cost of all sepsis survivors

**Fig. 1:**
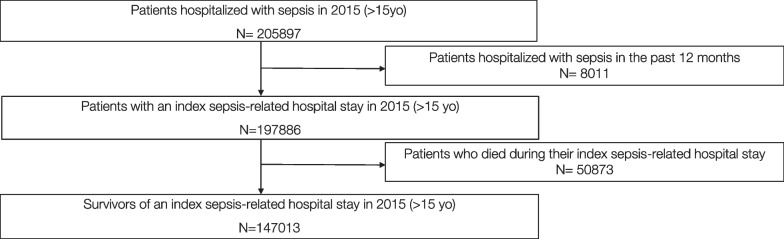
Flowchart of patient selection

Of 197,886 patients above 15 years old who had an index sepsis-related hospitalization in France in 2015, 147,013 (74.3%) survived their hospitalization and were included in our cohort (Fig. 1). Of these survivors, 57.1% were men, 60.4% were aged over 65 years, and 27.3% had a Charlson score > 2 (Table 1). Their cumulative mortality reached 36.6% at 3 years post-sepsis. However, the yearly mortality rate among survivors declined from 22.9 to 8.4%, at 1 and 3 years post-sepsis, respectively (See Additional file 1: Fig. S1). Most survivors (89.8%) had medical impairment at baseline, yet 79.2% developed new medical impairment in the first year post-sepsis. Similarly, 37.0% and 25.5% of survivors, respectively, had psychological or cognitive impairments at baseline, while 26.9% and 18.5% developed new psychological or cognitive impairments in the first-year post-sepsis. The incidence of long-term ventilation (2.1%) and dialysis (2.4%) post-sepsis was, respectively, around three times (6.1%) and five times (12.2%) higher in populations at high risk (See Additional file 1: Table S3).

**Table 1:** Patients and index hospital stay characteristics of all survivors and by cluster

	All survivors	Cluster 1	Cluster 2	Cluster 3	Cluster 4	Cluster 5
		Early death	Late death	Short-term rehabilitation	Long-term rehabilitation	Home
	*N* = 147,013 (100%)	*N* = 19,003 (12.9%)	*N* = 10,058 (6.8%)	*N* = 16,597 (11.3%)	*N* = 4865 (3.3%)	*N* = 96,490 (65.6%)
*Patient characteristics, %*^a^						
Sex						
Men	57.1	59.0	60.0	53.8	62.2	56.7
Women	42.9	41.1	40.0	46.2	37.8	43.3
Age, median [IQR^a^]	70 [58–81]	77 [65–85]	75 [64–84]	75 [64–83]	64 [52–75]	68 [55–79]
16–30	4.0	0.5	0.8	2.1	6.7	5.2
31–45	7.0	2.3	3.1	3.9	9.9	8.8
46–55	9.9	6.2	6.9	7.5	13.9	11.2
56–65	18.7	16.0	17.9	15.2	22.7	19.6
66–75	22.1	21.6	22.6	22.3	22.8	22.1
76–85	24.8	30.0	28.8	32.3	17.8	22.5
> 85	13.5	23.4	19.8	16.8	6.3	10.6
Charlson, median [IQR^a^]	2 [0–3]	2 [2–6]	2 [2–5]	2 [0–3]	2 [0–2]	1 [0–2]
0	36.2	16.4	17.4	34.8	31.0	42.6
1–2	36.4	34.1	37.0	39.2	44.8	35.9
3–4	14.3	18.0	19.2	17.1	15.7	12.5
≥ 5	13.0	31.5	26.4	8.9	8.6	8.9
*Incident hospital stay characteristics, %*^a^						
Length of stay (days), median [IQR^2^]	16 [10–28]	19 [11–31]	17 [10–28]	27 [17–43]	38 [21–62]	14 [9–23]
< 7	12.0	10.3	10.3	3.3	3.6	14.5
7–30	66.2	63.7	67.8	54.4	35.0	70.1
31–90	20.1	24.2	20.4	38.7	49.9	14.5
> 90	1.7	2.0	1.5	3.7	11.5	0.9
ICU^c^ admission	56.8	44.2	47.4	72.6	79.7	56.4
Septic shock	16.4	16.7	15.5	22.3	22.3	15.0
Hospital discharge						
Acute care	15.4	21.1	12.9	25.8	28.5	12.1
Home	63.2	49.7	67.1	12.8	14.0	76.6
Home care	1.1	3.2	1.6	0.2	0.4	0.8
Long term care	20.3	26.1	18.5	61.2	57.2	10.5

^a^For each variable: percentage of patients for each cluster and all survivors

^b^Interquartile range

^c^Intensive care unit

Inpatient hospitalization among survivors was greater in the year post-sepsis than the year pre-sepsis (Table 2), while visits to ambulatory care were relatively stable (See Additional file 1: Table S4). In particular, inpatient hospitalization in acute care increased from 59.9 to 65.3% of patients, inpatient hospitalization in a rehabilitation facility from 11.8 to 33.8%, and hospital-at-home care from 2.2 to 7.7% (Table 2). While patients spent most of their time at home in the year post-sepsis, they spent more than half of their days available with nursing care or physiotherapy (See Additional file 1: Table S5). However, the percentage of survivors with inpatient hospitalization in both acute care (65.3–37.4%) and rehabilitation facilities (33.8–8.1%) declined annually over the 3 years post-sepsis, as did the percentage with hospital outpatient visits (78.9–64.8%), nursing care and physiotherapy (67.3–45.7%). Conversely, the share of patients with ambulatory visits to general practitioners and specialists remained stable over the 3 years post-sepsis (See Additional file 1: Fig. S2).

**Table 2:** Proportion of patients hospitalized in acute care, rehabilitation, hospital-at-home and psychiatry for all survivors and each cluster, in the 1-year period pre- and post-sepsis

	All survivors		Cluster 1		Cluster 2		Cluster 3		Cluster 4		Cluster 5	
			Early death		Late death		Short-term rehabilitation		Long-term rehabilitation		Home	
*Hospitalization 1-year pre-sepsis*												
Inpatient hospitalization in acute care												
Patients *N* %	88,052	59.9	14,306	75.3	7503	74.6	9804	59.1	2579	53.0	53,860	55.8
Days, median [IQR]^a^	14 [0–28]		20 [2–26]		19 [3–35]		13 [0–28]		16 [0–35]		11 [0–24]	
Day care in acute care												
Patients *N* %	52,958	36.0	8911	46.9	4850	48.2	4663	28.1	1255	25.8	33,279	34.5
Days, median [IQR]^a^	2 [1–8]		5 [1–17]		5 [1–15]		1 [1–3]		1 [1–3]		2 [1–6]	
Inpatient hospitalization in rehabilitation												
Patients *N* %	17,304	11.8	3533	18.6	1592	15.8	3408	20.5	1033	21.2	7738	8.0
Days, median [IQR]^a^	30 [3–70]		33 [3–91]		31 [3–60]		36 [8–74]		35 [8–89]		29 [3–66]	
Day care in rehabilitation												
Patients *N* %	1669	1.1	158	0.8	95	0.9	239	1.4	98	2.0	1079	1.1
Days, median [IQR]^a^	30 [18–55]		32 [18–57]		32 [19–57]		31 [17–58]		43 [22–85]		29 [18–50]	
Hospital-at-home												
Patients *N* %	3236	2.2	1039	5.5	382	3.8	170	1.0	120	2.5	1525	1.6
Days, median [IQR]^a^	32 [14–73]		34 [13–73]		29 [13–68]		33 [15–65]		30 [15–85]		32 [14–76]	
Psychiatry												
Patients *N* %	3150	2.1	265	1.4	175	1.7	363	2.2	164	3.4	2183	2.3
Days, median [IQR]^a^	37 [15–104]		34 [13–94]		42 [18–122]		36 [18–81]		45 [16–139]		37 [15–106]	
*Hospitalization 1-year post-sepsis*												
Inpatient hospitalization in acute care												
Patients *N* %	96,061	65.3	13,175	69.3	9042	89.9	11,904	71.7	3881	79.8	58,059	60.2
Days, median [IQR]^a^	17 [0–35]		16 [0–29]		32 [9–57]		21 [0–40]		28 [3–57]		14 [0–31]	
Day care in acute care												
Patients *N* %	52,948	36.0	4030	21.2	5030	50.0	5544	33.4	1822	37.5	36,522	37.9
Days, median [IQR]^a^	2 [1–9]		2 [1–5]		5 [1–12]		2 [1–7]		2 [1–7]		2 [1–10]	
Inpatient hospitalization in rehabilitation												
Patients *N* %	49,688	33.8	6355	33.4	3644	36.2	16,597	100	4865	100	18,227	18.9
Days, median [IQR]^a^	35 [21–68]		24 [11–43]		40 [22–70]		53 [37–76]		177 [138–243]		22 [16–29]	
Day care in rehabilitation												
Patients *N* %	5238	3.6	34	0.2	73	0.7	1502	9.1	1033	21.2	2596	2.7
Days, median [IQR]^a^	43 [19–81]		19 [8–34]		32 [2–72]		52 [26–88]		73 [30–136]		33 [16–60]	
Hospital-at-home												
Patients *N* %	11,356	7.7	2855	15.0	1732	17.2	837	5.0	459	9.4	5473	5.7
Days, median [IQR]^a^	29 [12–69]		19 [8–40]		35 [12–97]		41 [18 – 79]		73 [32–145]		32 [14–77]	
Psychiatry												
Patients *N* %	3168	2.2	81	0.4	120	1.2	390	2.4	146	3.0	2431	2.5
Days, median [IQR]^a^	40 [17–108]		19 [8–47]		33 [13–113]		42 [21–88]		58 [21–171]		41 [17–112]	
One-year mortality	33,608	22.9	19,003	100	10,058	100	696	4.2	336	6.9	3515	3.6
No. days of survival ^b^ median [IQR]	366 [365–366]		37 [15–68]		183 [144–228]		366 [366–366]		366 [366–366]		366 [366–366]	
No. days at home^b^ median [IQR]	328 [187–360]		2 [0–23]		113 [74–159]		284 [247–310]		108 [28–169]		352 [328–365]	

^a^Only among patients hospitalized in the medical unit of interest. *IQR* interquartile range

^b^In the following year only

The median cost per patient increased by 1473€ for hospitalization in acute care, by 311€ for hospitalization in rehabilitation, by 672€ for ambulatory visits and by 78€ for outpatient hospital visits post-sepsis. (Mean cost is also available in Additional file 1: Table S6.) Compared to the costs of pre-sepsis care (2.5 billion €), the total medical cost (ambulatory care and hospitalization in acute care or rehabilitation) per patient was higher post-sepsis, reaching 3.4 billion €, giving an increase of 856 million € in the year post-sepsis (See Additional file 1: Table S7). Hospitalization in acute care post-sepsis reached 1.5 billion € post-sepsis. In the year post-sepsis, the median cost of hospitalization in acute care was twice higher than the median cost of ambulatory care and the total cost of acute care was three times higher than the total cost of rehabilitation (See Additional file 1: Table S7). The medical cost of care tended to decline over the 3 years post-sepsis (See Additional file 1: Table S8).

### Patient characteristics, sequels and healthcare use and cost according to sepsis survivor profiles

#### State sequence analyses: identification of sepsis survivor profiles

**Fig. 2:**
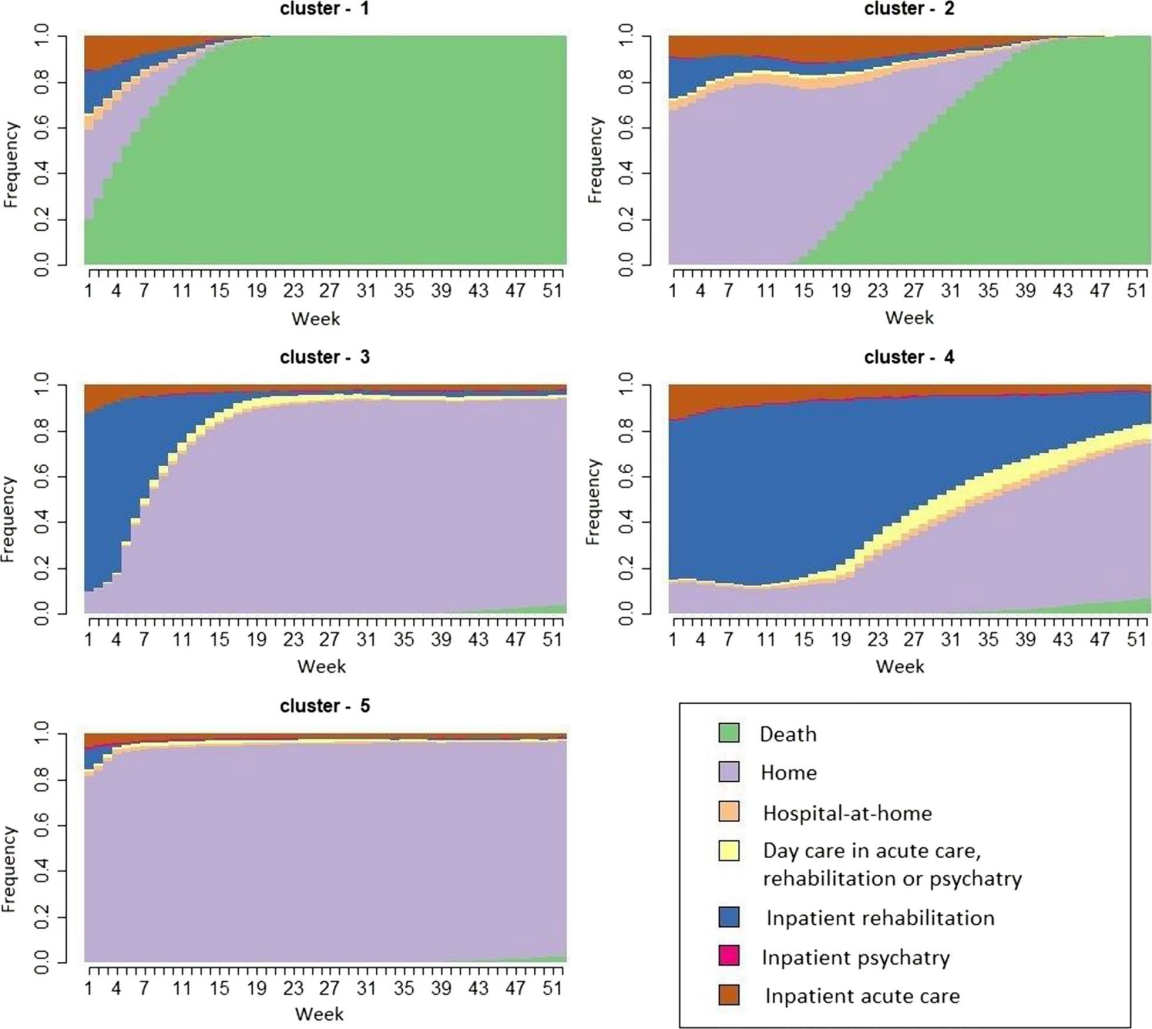
Results of the state sequence analysis of the 1-year post-sepsis period: weekly distribution of the health states by cluster. This figure is composed of 5 chronograms for each of the 5 identified care trajectories (clusters). On the *x* axis, time is graduated from discharge after the index sepsis hospitalization (week 1) to 1-year post-discharge (week 52). The *y* axis corresponds to the proportion of patients (from 0 to 1) in each health state. Clusters determined by the state sequence analysis of the healthcare pathways of survivors: cluster 1 (early death), cluster 2 (late death), cluster 3 (short-term rehabilitation), cluster 4 (long-term rehabilitation), cluster 5 (home)

After aggregating identical care pathway sequences, there were 43,693 distinct sequences across all sepsis survivors. Heterogeneity between sequences could be observed among all survivors (See Additional file 1: Fig. S3). The clustering analyses allowed to sort out differences between sequences and sepsis survivors were categorized into 5 clusters, reflecting the dominant characteristics of their care pathways in the year following their index sepsis hospitalization. These included: cluster 1: early death (~ ≤ 3 months) (19,003 patients, 12.9%); cluster 2: late death (~ ≥ 3 months) (10,058 patients, 6.8%); cluster 3: short-term rehabilitation (~ ≤ 3 months) before returning home (16,597 patients, 11.3%); cluster 4: long-term rehabilitation (~ > 3 months) (4865 patients, 3.3%); and 5) home (96,490 patients, 65.6%) (Fig. 2 and see Additional file 1: Fig. S4a, b). The clustering analysis provided a reasonable split between clusters (Average Silhouette Width of 0.51). The mean transversal entropy, which is a measure of the diversity of states within a cluster, was relatively low for all clusters (0.16), but higher for late death (0.42) and long-term rehabilitation clusters (0.46), reflecting greater heterogeneity in these clusters (See Additional file 1: Fig. S4c, d). However, in the last weeks of the care pathway, heterogeneity was only observed in the long-term rehabilitation cluster.

#### Characteristics of patients and their index hospital stays in each cluster

Relative to all survivors, men were overrepresented in the long-term rehabilitation cluster (62.2%) and underrepresented in the short-term rehabilitation cluster (53.8%). Patients were younger in the home (median 68 years) and long-term rehabilitation (64 years) clusters than those in the early death (77 years), late death (75 years) and short-term rehabilitation (75 years) clusters (Table 1). The proportion of patients with a Charlson index above 2 was highest in early death (49.5%) and late death (45.6%) clusters and lowest in the home cluster (21.5%). Two comorbidities were markedly more prevalent in some clusters compared to others: cancer in early death (45.1%) and late death (44.2%) clusters, and paraplegia/hemiplegia in the long-term rehabilitation cluster (29.4%) (Table 3).

**Table 3:** Percentage of patients with comorbidities across all survivors and in each cluster

Comorbidities	All survivors	Cluster 1	Cluster 2	Cluster 3	Cluster 4	Cluster 5
Heart failure (%)	20.4	24.5	24.7	26.2	17.4	18.4
Dementia (%)	5.3	10.3	8.6	4.8	2.9	4.2
Chronic pulmonary disease (%)	11.9	12.1	13.3	13.2	9.2	11.6
Rheumatologic disease (%)	1.3	1.2	1.0	1.6	1.5	1.3
Liver disease (%)	5.5	6.0	6.4	5.2	4.6	5.4
Diabetes with chronic complications (%)	6.4	6.5	6.8	7.7	10.7	6.0
Paraplegia and hemiplegia (%)	5.2	5.8	4.1	9.4	29.4	3.3
Renal disease (%)	12.6	15.5	15.7	13.1	11.2	11.6
Cancer (%)	23.2	45.1	44.2	15.4	12.9	18.5
AIDS, HIV (%)	0.5	0.3	0.4	0.5	0.8	0.6

Clusters determined by the state sequence analysis of the healthcare pathways of survivors: cluster 1 (early death), cluster 2 (late death), cluster 3 (short-term rehabilitation), cluster 4 (long-term rehabilitation), cluster 5 (home)

The median length of the index hospital stay was markedly higher (27 and 38 days, respectively) for patients having subsequent short- or long-term rehabilitation (cluster 3 and 4) compared to the overall population (16 days). The proportion of patients with a length of stay > 30 days reached 42.3% and 61.4% for clusters 3 and 4, respectively, with patients in the latter cluster having a higher proportion (11.5%) of very long stay (> 90 days) (Table 1). Patients in these two clusters also had the highest proportion of ICU admission and septic shock. Multiple infection sites were more common in cluster 3 and 4 (31.3% and 36.4% respectively). Compared to other clusters, primary bacteremia and heart and mediastinum infection were slightly more frequent in early death cluster (cluster 1) (22.3%; 6.2%) and 2 (22.1%; 7.1%) (See Additional file 1: Fig. S5).

#### Healthcare use and cost pre- and post-sepsis in each cluster

As expected, day care and inpatient care in rehabilitation facilities increased in short-term and long-term rehabilitation clusters (Table 2). However, compared to long-term rehabilitation cluster, patients in short-term rehabilitation cluster had a high proportion of time available spent at home (73.9%) (See Additional file 1: Table S5). The share of patients receiving hospital-at-home care was higher in the early death and late death clusters pre-sepsis and markedly increased in the year post-sepsis (15.0% and 17.2% respectively). These patients also had the highest percentage of patients in day care or fully hospitalized in acute care in the 1-year period post-sepsis (50% and 89.9% respectively) (Table 2). Indeed, patients in early and late death cluster spent the highest proportion of their time alive as inpatient hospitalized in acute care (37% and 20.2% respectively) (See Additional file 1: Table S5). Although patients in home cluster had low 1-year mortality rate (3.6%) and spent more time at home post-sepsis than other clusters, they nonetheless experienced a doubling in the share of patients hospitalized in rehabilitation care (Table 2 and see Additional file 1: Table S5).

Compared to other clusters, those from the home cluster had a lower share of their time available (time when the patient was neither hospitalized nor dead) occupied by any form of ambulatory care (nursing care and physiotherapy, GP visits, specialist visits or hospital outpatient visits) (See Additional file 1: Table S5). An increase in the proportion of patients with hospital outpatient visits, as well as the number of visits per patients, was observed in short-term (pre: 72.8%; post: 89.4%) and long-term rehabilitation clusters and home cluster (pre: 75.5%; post: 84.1%) (See Additional file 1: Table S4) and inpatient hospitalization was higher compared to patients returning home over the next 3 years post-sepsis (See Additional file 1: Table S9). Despite spending a large share of their time available hospitalized in inpatient acute care (37.0%) and rehabilitation care (22.7%), patients in the early death cluster nonetheless spent a greater share of their time having GP visits (14.2%) than any other cluster (See Additional file 1: Table S5).

Differences for the costs of hospitalization and ambulatory care were identified between clusters. The median cost of the index hospitalization and the cost of ambulatory care and rehabilitation care during the year post-sepsis were higher in short and long-term rehabilitation clusters (See Additional file 1: Tables S6 and S7).

### Cognitive, psychological and medical impairment post-sepsis in each cluster

New medical impairments post-sepsis were particularly common for patients from late death cluster, and short-term and long-term rehabilitation clusters (91.2%, 92.8% and 94.8% respectively) (See Additional file 1: Table S3). New psychological impairments were identified in almost half of the patients in long-term rehabilitation cluster (46.3%). The highest incidence of new cognitive impairments was in short- and long-term rehabilitation clusters (30.5% and 33.9% respectively) (See Additional file 1: Table S3 and Fig. S6).

## Discussion

This study allows to identify different patient profiles, based on their care pathway, with different mortality rate, sequels and healthcare usage over the year prior and three years subsequent to their index episode of sepsis.

### Consequence for all survivors: long-term mortality, health impairment and care consumption and cost

In line with the estimates from previous studies, 36.6% of patients who survive an index sepsis hospitalization die within three years and sepsis survivors showed a high rates of post-sepsis sequels, including new medical (79.2%), psychological (26.9%) and cognitive (18.5%) impairments [3, 18, 28]. Hospitalization and ambulatory care, indicators of the health status, tended to increase post-sepsis, especially hospitalization in rehabilitation facilities [3, 17, 17, 29]. In line with several publications, the proportion of patients with nursing care/physiotherapy, specialist visits and outpatient visits increased in the first year post-sepsis but declined in the following years [30–33]. This could reflect less healthcare dependency, but also less than optimal sepsis aftercare for some patients [30, 31, 34]. In line with a recent study, rehospitalization in acute care declined but remained above 30% in the third year post-sepsis [30]. Indeed, sepsis patients, due to preexisting comorbidities and immune, physical or cognitive impairments, are extremely vulnerable and at high risk of hospital readmission for sepsis itself or other causes [15, 29, 35]. Prescott and Angus suggest that rehospitalization post-discharge might be partially treatable in the outpatient setting, possibly through better interactions between hospitalization and ambulatory care and a care pathway adapted to each patient profile [31]. The median medical cost of the index sepsis-related hospitalization was slightly lower compared to previous studies conducted in Japan, the USA and France but the cost of hospitalization in the year post-sepsis was in line with studies in Germany [8, 30, 34, 36, 37]. Discrepancy in the cost of hospitalization for sepsis could be explained by the difference in sepsis selection, the method to calculate costs and differences in healthcare systems, and possibly to the implementation of cost effectiveness strategies in hospital management [8, 37].

### Health impairment and healthcare consumption by patient profile

#### Patients with early and late 1-year death

Our results indicate that about 20% of sepsis survivors died within one year of their index sepsis episode (clusters 1 and 2), and that these patients tended to be older and have more comorbidities, especially cancer. Indeed, cancer but also cancer treatment are well known risk factors for sepsis and mortality, especially for the elderly [38–40]. A disproportionately large share of these patients had renal disease, chronic pulmonary disease and heart failure, which are also important risk factors for complications or death; new impairments post-sepsis might also have worsened their prognosis [3, 12, 29, 41–45]. These patients also experienced more hospital-at-home care post-sepsis than other clusters, which might have helped to limit further rehospitalization despite being unable to prevent death [40, 46, 47]. Patients with the earliest death spent more of their time spent at home with general practitioner visits, possibly associated with community-managed palliative care for some patients experiencing drastic declines [31, 48]. Additional studies are required to identify whether some of the late death in cluster 2 might have been preventable thanks to changes in post-discharge care, and also to assess the information flow and transition to palliative care when death was not preventable [17, 33, 48–50].

#### Patients with short- and long-term rehabilitation

Survivors in short-term and long-term rehabilitation clusters (clusters 3 and 4) represented about 15% of the survivors. The relatively high three-year mortality in these clusters is consistent with the high comorbidity indices and the high prevalence of multiple sites of infection during the index hospitalization [44, 45, 51]. Patients characteristics in one or both of these clusters like advanced age, a higher proportion of patients with septic shock, paraplegia and hemiplegia (possibly resulting from previous stroke), diabetes with complications, or requiring dialysis or long-term mechanical ventilation post-sepsis (as possible sequelae of acute kidney injury or respiratory distress syndrome), as well as greater psychological impairment associated with medical and cognitive impairment might have put those patients at higher risk of complication or death [28, 42, 44, 52–59]. Notwithstanding the relatively high mortality rate, further studies are required to assess the impact of rehabilitation on long-term survival and quality of life of these patients [28, 30, 31, 33, 34, 60, 61].

#### Patients returning home

Two-thirds of survivors, younger and with less comorbidities and fewer sequels, returned home after sepsis (cluster 5). Nevertheless, 73.1% of these patients developed a new medical impairment and 20% died in the 3 following years. The reduced amount of time occupied with nursing care/physiotherapy compared to other clusters could reflect better health status. However, substantial persisting health issues are likely with 60% having nursing and physiotherapy, specialist or hospital outpatient visits 1 year post-sepsis and 40% fully hospitalized in acute care. Moreover, hospital readmission in acute care represented the most important cost in the total care consumption. Further studies are required, to assess if changes in short and long-term aftercare, including rehabilitation, advanced hospital-at-home care, specific post-sepsis follow-up or patient education initiatives to improve the transfer of critical medical information, could possibly reduce mortality or rehospitalization and its associated cost [30, 31, 34, 46, 47, 60, 62]. Indeed, a study conducted in Germany showed better 5-year survival for a group of patients transferred to rehabilitation compared to a similar group of patients discharged home or to self-care [34].

#### Limitations

This study is based on a secondary analysis of medical administrative data, and it was not possible to validate sepsis diagnoses using clinical data. Moreover, this analysis was limited to sepsis of presumed bacterial etiology. However, previous analyses have demonstrated the robustness of the selection algorithm used in this work, with a reasonable representativeness of all sepsis cases [5, 63]. Another limitation is that the available variable in our database used to calculate the cost of rehabilitation tends to overestimate these costs compared to a newer variable implemented in 2017. Since our data collection began in 2014, the cost of rehabilitation should be estimated again in future studies after 2017, and present results should be interpreted with caution. Finally, working with secondary data, this cohort study could not prove causal relationship but provides a holistic approach to the study of care pathway and interesting information to build further studies on specific patient profiles.

## Conclusion

We have estimated high rates of post-sepsis mortality and sequels over 3 years as well as an increased usage and cost of healthcare services. Yet behind these general trends, our clustering analysis identified heterogeneous trajectories of care after sepsis allowing to identify various patient profiles. This analysis highlighted the high cost of rehospitalization post-sepsis, moderate use of rehabilitation facilities and the high percentage of patients returning home with non-negligible health impairments, questioning whether sepsis aftercare could be improved. Several publications suggest that some of the deaths or rehospitalizations might be preventable. Targeted interventions and personalization of the healthcare pathway according to patient profile, in order to improve survival and quality of life and cost effectiveness of the healthcare system, should be further studied. Notwithstanding the persistently high death rate post-sepsis, more than 60% of the survivors were still alive after 3 years, most of them with new medical, cognitive or psychological impairments. This highlights the importance to conduct further studies on short- and long-term post-discharge care needs to reduce the sequels of sepsis.

### Supplementary Information

**Additional file 1: Methods.** Databases and Methods, **Table S1.** ICD-10 codes used to identify sepsis of presumed bacterial etiology according to type of selection in sepsis patients > 15 years. **Table S2** Description of the variables. **Fig. S1.** Number of patients and percentage of deaths in the 3 consecutive years post-sepsis (2016–2018). **Table S3.** Cognitive, psychological, and medical impairment for sepsis survivors in the 1-year period following the index sepsis episode. **Table S4.** Proportion of patients with ambulatory care, for each cluster, in the 1-year period pre- and post-sepsis and number of visits. **Table S5.** Mean number of ambulatory visits and mean time spent hospitalized or at home, during the year pre- and post-sepsis, for each cluster. **Fig. S2.** Percentage of surviving patients with hospitalizations and ambulatory visits among sepsis survivors in the 3 consecutive years following their index sepsis hospitalization. **Table S6.** Mean and 95% confidence interval [95CI] of the cost in euros per patient for the index hospitalization and the 1-year period pre- and post-sepsis care. **Table S7.** Global cost of sepsis and median and interquartile cost per patient for the index hospitalization and the 1-year period pre- and post-sepsis care. **Table S8.** Total and median [IQR] cost in euros per survivor of ambulatory care and hospitalization during 2nd and 3rd year post-sepsis. **Fig. S3.** Visualization of all sequences without clustering. **Fig. S4.** Results of the Sequences and the clustering analyses. **Fig. S5.** Distribution of the infection sites across all survivors and in each cluster. **Table S9.** Long-term assessment of hospitalization, mortality and ambulatory visits (2015–2018). **Fig. S6.** Prevalent (in gray in the figure) and incident (in red in the figure) medical, psychological and cognitive impairments in sepsis survivors in the 1-year period following the index sepsis episode.

## Data Availability

The data that support the findings of this study are available from the French administrative health care database (SNDS) but restrictions apply to the availability of these data, which were used under the French Data Protection Agency approval, and so are not publicly available.

## References

[1] WHO. WHA70.7, Agenda item 12.2. Improving the prevention, diagnosis and clinical management of sepsis. Published online May 29, 2017.

[2] Rudd KE, Johnson SC, Agesa KM (2020). Global, regional, and national sepsis incidence and mortality, 1990–2017: analysis for the Global Burden of Disease Study. Lancet.

[3] Fleischmann-Struzek C, Rose N, Freytag A (2021). Epidemiology and costs of postsepsis morbidity, nursing care dependency, and mortality in Germany, 2013 to 2017. JAMA Netw Open.

[4] Singer M, Deutschman CS, Seymour CW (2016). The third international consensus definitions for sepsis and septic shock (sepsis-3). JAMA.

[5] Pandolfi F, Guillemot D, Watier L, Brun-Buisson C (2022). Trends in bacterial sepsis incidence and mortality in France between 2015 and 2019 based on National Health Data System (Système National des données de Santé (SNDS)): a retrospective observational study. BMJ Open.

[6] Rhee C, Jentzsch MS, Kadri SS (2019). Variation in identifying sepsis and organ dysfunction using administrative versus electronic clinical data and impact on hospital outcome comparisons. Crit Care Med.

[7] Jolley RJ, Sawka KJ, Yergens DW, Quan H, Jetté N, Doig CJ (2015). Validity of administrative data in recording sepsis: a systematic review. Crit Care.

[8] Dupuis C, Bouadma L, Ruckly S (2020). Sepsis and septic shock in France: incidences, outcomes and costs of care. Ann Intensive Care.

[9] Farrah K, McIntyre L, Doig CJ (2021). Sepsis-associated mortality, resource use, and healthcare costs: a propensity-matched cohort study. Crit Care Med.

[10] Bauer M, Gerlach H, Vogelmann T, Preissing F, Stiefel J, Adam D (2020). Mortality in sepsis and septic shock in Europe, North America and Australia between 2009 and 2019- results from a systematic review and meta-analysis. Crit Care.

[11] Fay K, Sapiano MRP, Gokhale R (2020). Assessment of health care exposures and outcomes in adult patients with sepsis and septic shock. JAMA Netw Open.

[12] Prescott HC, Iwashyna TJ, Blackwood B (2019). Understanding and enhancing sepsis survivorship. Priorities for research and practice. Am J Respir Crit Care Med.

[13] Gadre SK, Shah M, Mireles-Cabodevila E, Patel B, Duggal A (2019). Epidemiology and predictors of 30-day readmission in patients with sepsis. Chest.

[14] Stenholt POO, Bin Abdullah SMO, Sorensen RH, Nielsen FE (2021). Independent predictors for 90-day readmission of emergency department patients admitted with sepsis: a prospective cohort study. BMC Infect Dis.

[15] Shankar-Hari M, Saha R, Wilson J (2020). Rate and risk factors for rehospitalisation in sepsis survivors: systematic review and meta-analysis. Intensive Care Med.

[16] Huang CY, Daniels R, Lembo A (2019). Life after sepsis: an international survey of survivors to understand the post-sepsis syndrome. Int J Qual Health Care J Int Soc Qual Health Care.

[17] Prescott HC, Langa KM, Liu V, Escobar GJ, Iwashyna TJ (2014). Increased 1-year healthcare use in survivors of severe sepsis. Am J Respir Crit Care Med.

[18] Winters BD, Eberlein M, Leung J, Needham DM, Pronovost PJ, Sevransky JE (2010). Long-term mortality and quality of life in sepsis: a systematic review. Crit Care Med.

[19] Liao TF, Bolano D, Brzinsky-Fay C (2022). Sequence analysis: Its past, present, and future. Soc Sci Res.

[20] Roux J, Grimaud O, Leray E (2019). Use of state sequence analysis for care pathway analysis: the example of multiple sclerosis. Stat Methods Med Res.

[21] Touat M, Brun-Buisson C, Opatowski M (2021). Costs and outcomes of 1-year post-discharge care trajectories of patients admitted with infection due to antibiotic-resistant bacteria. J Infect.

[22] Vogt V, Scholz SM, Sundmacher L (2018). Applying sequence clustering techniques to explore practice-based ambulatory care pathways in insurance claims data. Eur J Public Health.

[23] Vanasse A, Courteau J, Courteau M (2020). Healthcare utilization after a first hospitalization for COPD: a new approach of state sequence analysis based on the “6W” multidimensional model of care trajectories. BMC Health Serv Res.

[24] Angus DC, Linde-Zwirble WT, Lidicker J, Clermont G, Carcillo J, Pinsky MR (2001). Epidemiology of severe sepsis in the United States: analysis of incidence, outcome, and associated costs of care. Crit Care Med.

[25] Gabadinho A, Ritschard G, Müller NS, Studer M (2011). Analyzing and visualizing state sequences in R with TraMineR. J Stat Softw.

[26] Ioannidis JPA (2019). What Have we (not) learnt from millions of scientific papers with P values?. Am Stat.

[27] Lin M, Lucas HC, Shmueli G (2013). Too big to fail: large samples and the p-value problem. Inf Syst Res.

[28] Iwashyna TJ, Ely EW, Smith DM, Langa KM (2010). Long-term cognitive impairment and functional disability among survivors of severe sepsis. JAMA.

[29] Liu V, Lei X, Prescott HC, Kipnis P, Iwashyna TJ, Escobar GJ (2014). Hospital readmission and healthcare utilization following sepsis in community settings. J Hosp Med.

[30] Schmidt KFR, Huelle K, Reinhold T (2022). Healthcare utilization and costs in sepsis survivors in germany-secondary analysis of a prospective cohort study. J Clin Med.

[31] Prescott HC, Angus DC (2018). Enhancing recovery from sepsis. JAMA.

[32] Major ME, Kwakman R, Kho ME (2016). Surviving critical illness: what is next? An expert consensus statement on physical rehabilitation after hospital discharge. Crit Care.

[33] Gehrke-Beck S, Gensichen J, Turner KM, Heintze C, Schmidt KF (2021). General practitioners’ views and experiences in caring for patients after sepsis: a qualitative interview study. BMJ Open.

[34] Rahmel T, Schmitz S, Nowak H (2020). Long-term mortality and outcome in hospital survivors of septic shock, sepsis, and severe infections: the importance of aftercare. PLoS ONE.

[35] DeMerle KM, Royer SC, Mikkelsen ME, Prescott HC (2017). Readmissions for recurrent sepsis: new or relapsed infection?. Crit Care Med.

[36] Paoli CJ, Reynolds MA, Sinha M, Gitlin M, Crouser E (2018). Epidemiology and costs of sepsis in the United States—an analysis based on timing of diagnosis and severity level*. Crit Care Med.

[37] Oami T, Imaeda T, Nakada T (2022). Temporal trends of medical cost and cost-effectiveness in sepsis patients: a Japanese nationwide medical claims database. J Intensive Care.

[38] Hensley MK, Donnelly JP, Carlton EF, Prescott HC (2019). Epidemiology and outcomes of cancer-related versus non-cancer-related sepsis hospitalizations. Crit Care Med.

[39] Liu Z, Mahale P, Engels EA (2019). Sepsis and risk of cancer among elderly adults in the United States. Clin Infect Dis.

[40] Abou Dagher G, El Khuri C, Chehadeh AAH (2017). Are patients with cancer with sepsis and bacteraemia at a higher risk of mortality? A retrospective chart review of patients presenting to a tertiary care centre in Lebanon. BMJ Open.

[41] Yang WS, Kim YJ, Ryoo SM, Kim WY (2021). Independent risk factors for sepsis-associated cardiac arrest in patients with septic shock. Int J Environ Res Public Health.

[42] Doi K, Leelahavanichkul A, Hu X (2008). Pre-existing renal disease promotes sepsis-induced acute kidney injury and worsens sepsis outcome via multiple pathways. Kidney Int.

[43] Dagher GA, Hajjar K, Khoury C (2018). Outcomes of patients with systolic heart failure presenting with sepsis to the emergency department of a tertiary hospital: a retrospective chart review study from Lebanon. BMJ Open.

[44] Esper AM, Moss M, Lewis CA, Nisbet R, Mannino DM, Martin GS (2006). The role of infection and comorbidity: factors that influence disparities in sepsis. Crit Care Med.

[45] Shankar-Hari M, Ambler M, Mahalingasivam V, Jones A, Rowan K, Rubenfeld GD (2016). Evidence for a causal link between sepsis and long-term mortality: a systematic review of epidemiologic studies. Crit Care.

[46] Paulson MR, Torres-Guzman RA, Matcha GV (2023). Treatment of a high healthcare utilizer with sepsis in a virtual hybrid hospital-at-home program. Clin Case Rep.

[47] Thursky K, Lingaratnam S, Jayarajan J (2018). Implementation of a whole of hospital sepsis clinical pathway in a cancer hospital: impact on sepsis management, outcomes and costs. BMJ Open Qual.

[48] Reymond L, Parker G, Gilles L, Cooper K (2018). Home-based palliative care. Aust J Gen Pract.

[49] Cousin F, Gonçalves T, Dauchy S, Marsico G. Atlas des soins palliatifs et de la fin de vie en France: Troisième édition - 2023. Published online March 1, 2023. https://www.google.com/url?sa=t&rct=j&q=&esrc=s&source=web&cd=&ved=2ahUKEwj_6Mq93uX_AhV6U6QEHTfJCkEQFnoECAwQAQ&url=https%3A%2F%2Fwww.parlons-fin-de-vie.fr%2Fwp-content%2Fuploads%2F2023%2F03%2Fatlas-2023.pdf&usg=AOvVaw3w9zyTpXBkWIuit2yqXQUs&opi=89978449

[50] Cecconi M, Evans L, Levy M, Rhodes A (2018). Sepsis and septic shock. Lancet.

[51] Chou EH, Mann S, Hsu TC (2020). Incidence, trends, and outcomes of infection sites among hospitalizations of sepsis: a nationwide study. PLoS ONE.

[52] Lee SH, Hsu TC, Lee MTG (2018). Nationwide trend of sepsis: a comparison among octogenarians, elderly, and young adults. Crit Care Med.

[53] Sehgal V, Bajwa SJS, Consalvo JA, Bajaj A (2015). Clinical conundrums in management of sepsis in the elderly. J Transl Intern Med.

[54] Frydrych LM, Fattahi F, He K, Ward PA, Delano MJ (2017). Diabetes and sepsis: risk, recurrence, and ruination. Front Endocrinol.

[55] Stösser S, Isakeit J, Bode FJ, Bode C, Petzold GC (2022). Sepsis in patients with large vessel occlusion stroke-clinical characteristics and outcome. Front Neurol.

[56] Peerapornratana S, Manrique-Caballero CL, Gómez H, Kellum JA (2019). Acute kidney injury from sepsis: current concepts, epidemiology, pathophysiology, prevention and treatment. Kidney Int.

[57] Hu Q, Hao C, Tang S (2020). From sepsis to acute respiratory distress syndrome (ARDS): emerging preventive strategies based on molecular and genetic researches. Biosci Rep.

[58] Nikayin S, Rabiee A, Hashem MD (2016). Anxiety symptoms in survivors of critical illness: a systematic review and meta-analysis. Gen Hosp Psychiatry.

[59] Rabiee A, Nikayin S, Hashem MD (2016). Depressive symptoms after critical illness: a systematic review and meta-analysis. Crit Care Med.

[60] Winkler D, Rose N, Freytag A (2023). The effects of postacute rehabilitation on mortality, chronic care dependency, health care use, and costs in sepsis survivors. Ann Am Thorac Soc.

[61] Schmidt K, Thiel P, Mueller F (2014). Sepsis survivors monitoring and coordination in outpatient health care (SMOOTH): study protocol for a randomized controlled trial. Trials.

[62] Auerbach AD, Kripalani S, Vasilevskis EE (2016). Preventability and causes of readmissions in a national cohort of general medicine patients. JAMA Intern Med.

[63] Pandolfi F, Brun-Buisson C, Guillemot D, Watier L (2022). One-year hospital readmission for recurrent sepsis: associated risk factors and impact on 1-year mortality-a French nationwide study. Crit Care.

