# Supplementary material for: Care pathways of sepsis survivors: sequelae, mortality and use of healthcare services in France, 2015–2018
Source: Crit Care. 2023 Nov 10;27:438. doi: 10.1186/s13054-023-04726-w (PMC10638811; doi:10.1186/s13054-023-04726-w)
Supplement: Supplementary file 1 — Additional file 1: Methods. Databases and Methods, Table S1. ICD-10 codes used to identify sepsis of presumed bacterial etiology according to type of selection in sepsis patients > 15 years. Table S2 Description of the variables. Fig. S1. Number of patients and percentage of deaths in the 3 consecutive years post-sepsis (2016–2018). Table S3. Cognitive, psychological, and medical impairment for sepsis survivors in the 1-year period following the index sepsis episode. Table S4. Proportion of patients with ambulatory care, for each cluster, in the 1-year period pre- and post-sepsis and number of visits. Table S5. Mean number of ambulatory visits and mean time spent hospitalized or at home, during the year pre- and post-sepsis, for each cluster. Fig. S2. Percentage of surviving patients with hospitalizations and ambulatory visits among sepsis survivors in the 3 consecutive years following their index sepsis hospitalization. Table S6. Mean and 95% confidence interval [95CI] of the cost in euros per patient for the index hospitalization and the 1-year period pre- and post-sepsis care. Table S7. Global cost of sepsis and median and interquartile cost per patient for the index hospitalization and the 1-year period pre- and post-sepsis care. Table S8. Total and median [IQR] cost in euros per survivor of ambulatory care and hospitalization during 2nd and 3rd year post-sepsis. Fig. S3. Visualization of all sequences without clustering. Fig. S4. Results of the Sequences and the clustering analyses. Fig. S5. Distribution of the infection sites across all survivors and in each cluster. Table S9. Long-term assessment of hospitalization, mortality and ambulatory visits (2015–2018). Fig. S6. Prevalent (in gray in the figure) and incident (in red in the figure) medical, psychological and cognitive impairments in sepsis survivors in the 1-year period following the index sepsis episode. [file 13054_2023_4726_MOESM1_ESM.docx]

**Additional file 1**

**Methods: Databases and Methods**

Databases: The study, analysis and data extraction were approved by the French Data Protection Agency (CNIL, approval DE-2016-176). Informed consent is waived for the use of these anonymised secondary data, as mentioned in the Social Security Code, Article L161–28-1. All methods were performed in accordance with CNIL regulations and with REporting of studies Conducted using Observational Routinely collected Data (RECORD) guideline. The National Health Data System (**Système National des Données de Santé:** SNDS) essentially contains individual data used for billing and reimbursement of outpatient health care consumption (Inter-Scheme consumption data: DCIR) and private and public hospital data (Medical Information System Programme: PMSI) collected by the Agence Technique de l’Information sur l’Hospitalisation (ATIH)**^1^***.*  These pseudonymized patient-level data include all inpatient and outpatient visits and ambulatory care received in the community, including visits to general practitioners, specialists, and nursing and physiotherapy care (including speech therapists).

Sepsis selection: Regarding sepsis selection in the PMSI, sepsis was identified as the combination of explicit and implicit sepsis. The selection of septic patients in the medico-administrative database is based on a translation of the Sepsis-3 definition**^2^**. Explicit sepsis was defined as a stay for which sepsis explicitly appeared in the ICD-10 codes (e.g., A40: streptococcal sepsis) as primary diagnosis (PD: condition requiring hospitalization), related diagnosis (RD: adds information to PD) or significant associated diagnosis (SAD: complications and co-morbidities potentially affecting the course or cost of hospitalization). In the absence of specific sepsis ICD-10 codes, implicit sepsis was defined as a stay with one of the selected ICD-10 codes for infection as PD, RD or SAD, with two associated conditions: ICU admission and one of the selected ICD-10 codes for organ dysfunction or a code for organ support from the Common Classification of Medical Acts (CCAM). The lists of ICD-10 codes and codes for organ dysfunction or support were established according to previous publications and based on medical and epidemiological expertise.

Hospital stays identification: The index sepsis-related hospital stays and a maximum of two subsequent sepsis-related hospital stays were grouped together and considered as a unique index hospital stay when:

- the interval between discharge and the subsequent admission was <1day

or

- between 1 and 7 days elapsed between discharge and the subsequent admission, and the subsequent hospital stay had the same primary diagnosis or bacteremia as primary diagnosis

For the sepsis-related hospital stays grouped together as a unique hospital stay, death, ICU admission and septic shock were considered if recorded during any one of these hospital stays. The admission date of the first stay and the discharge date of the last stay were considered. The lengths of stay were re-calculated considering possible overlap. Age, gender, comorbidities and infection site of the index sepsis-related hospitalization were considered.

State sequence analysis (SSA) and clustering: After building the sequence based on the 7 possible states *(death of the patient (DEAD); inpatient hospitalization in an acute care facility (MSO_full), a rehabilitation facility (REHAB_full), or a psychiatric facility (PSY_full); hospital-at-home (HAH);* *day care in an acute care, rehabilitation or psychiatric facility (DAYCARE); and staying at home with or without ambulatory care (HOME))* , if several states were identified within the same time unit, priority was given to DEATH, then to MSO_full, followed by REHAB_full, PSY_full, HAH, DAYCARE and finally HOME. In total, all sequences contain 7931507 time units; including 12710 (0.16%) time units with overlapping states and for which a unique corresponding state was selected based on the priority mentioned above. In total, 3% of the patients had overlap in their sequences. For the SSA, Optimal matching was used to estimate the distance between sequences with an insertion/deletion and substitution cost derived from the observed transition rates^3–5^. The distance matrix was further used to cluster the different sequences based on two complementary methods: Ascendant Hierarchical Clustering based on Ward method (AHC) and Partitioning Around Medoids (PAM). The optimal clustering methods and the interpretation of the results was based on Average Silhouette Width (ASW), the transversal entropy and the visualization of states sequences plots (Plot the state distribution, plot of the sequence of modal states, plot of the mean time spent in each state, plot of transversal entropies and index plot). Due to the high number of patients, identical sequences were first aggregated to reduce the memory and the computing time used^6^. The sequences were further disaggregated to attribute a cluster to each patient and produce the different plots.

Calculation of the ambulatory visits and hospitalization ratios: Because the survival time and hospitalization length after the index sepsis-related hospitalization differed between patients, ambulatory visits and hospitalization (inpatient or day care) were also expressed as ratio in the 12 months following the index sepsis episode. First, the number of days available in the following 12 months was assessed for each patient (days where the patient is neither dead or hospitalized in hospital-at-home, acute care, rehabilitation or psychiatric facility). Secondly, the number of days of ambulatory visits, including hospital outpatient visits, were also assessed for each patient. As a result, the ratio between the number of days occupied by ambulatory visits and days available was calculated. Similar methodology was used to assess the ratio between the number of days of hospitalization in hospital-at-home, acute care, rehabilitation or psychiatric facility and the days available in the following 12 months (= number of days where the patient is alive).

Cognitive, psychological and medical impairment

We used the methods of Fleischmann *et al.*^7^ to identify cognitive, medical and psychological impairment. The morbidity was considered if one of the relevant ICD-10 code was reported during at least one hospitalization or one hospital outpatient or ambulatory visits in the 1-year period following the index episode of sepsis. This was translated in the French medico-administrative database as one ICD-10 as:

- Primary diagnosis, related diagnosis or significant associated diagnosis recorded in acute facilities (Medicine, surgery, obstetric)
- Main morbid symptom, etiological diagnosis, significant associated diagnosis or the main purpose of care recorded in rehabilitation
- Primary diagnosis in home care or significant associated diagnosis recorded in hospital-at-home
- Primary diagnosis in home care or significant associated diagnosis recorded in psychiatry
- Long-term medical condition or pathology recorded during ambulatory visits

| **Impairment** | **ICD-10 Codes** |
| --- | --- |
| - **Cognitive** | F00, F01, F02, F03, F04, F05, F067, F069, F078, F079, G30, G310, G311, G319, G32, R410, U51 |
| - **Psychological** |  |
| PTSD* | F43, F430, F431, F432, F438, F439 |
| Depression | F063, F32, F33, F341, F38, F412 |
| Anxiety | F064, F40, F41 |
| Sleeping disorders | F51, G47 |
| Substance abuse | F10, F11, F12, F13, F14, F15, F16, F17, F18, F19 |
| **Medical** |  |
| Respiratory dysfunction | J80, J96, J98, R060 |
| Cardiovascular diseases | I20, I22, I24, I25, I42, I47, I48, I49, I50 |
| Cerebrovascular diseases | I63, I64, I65, I66 |
| Renal diseases | N17, N18, N19 |
| Hepatic diseases | K721 |
| Metabolic diseases | E11, E12, E13, E14, E23, E27, E349, E35 |
| Anemia | D50, D51, D52, D53, D63, D649 |
| Neuromuscular/musculoskeletal diseases | G628, G728, M2162, M245, M256, M624, R13, R262, R263, R296, R49, Z740, Z993 |
| Decubitus | L89 |
| Complications of tracheostomy | J386, J398, J950, J955, J9581, Z430, Z930 |
| Urogenital diseases | F52, N393, N394, N991, R15, R32 |
| Sensory disorders | H90, H91, H93, R42, R43 |
| Impairment of nutrition | E41, E43, E44, E46, R630, R633, R634, R636, R638, R64 |
| Multidrug-resistant infections | U80, U81, U82, U83, U84 |
| Chronic pain | F454, G546, R521, R522, R529 |
| Fatigue | G933, R53 |

* Post-traumatic stress disorder

Similarly, Dialysis was identified if one of the relevant ICD-10 code or CCAM (**Common Classification of Medical Acts)** was reported during at least one hospitalization (in patient or day care) or one hospital outpatient or ambulatory *visits* in the 1-year period following the index episode of sepsis. CCAM codes are an adaptation of the International Classification of Procedures in Medicine, corresponding to the OPS used by Fleischmann *et al.*. Long-term mechanical ventilation was identified if one of the relevant ICD-10 code or CCAM procedure was reported during the time period between 3 months and 12 months post-sepsis, in at least one hospitalization or one hospital outpatient or ambulatory visits. A delay of 3 months was applied, not to include possible weaning from mechanical ventilation, which was not considered as long-term mechanical ventilation. The population at risk of long-term mechanical ventilation included the patients with chronic respiratory disease and without mechanical ventilation in the 1-year period preceding the index sepsis episode. Chronic respiratory disease was considered if one of the following ICD-10 codes was reported during at least one hospitalization or one hospital outpatient or ambulatory visits in the 1-year period preceding the index episode of sepsis: B909, C33, C34, C450, C38, C39, E662, G473, J684, I270, I272, I279, I289, J41, J42, J43, J44, J45, J46, J47, J60, J61, J62, J63, J64, J65, J66, J67, J701, J703, J82, J84, J92, J953, J961, J969, J980, J982, J983, J984, J985, J986, J989, J99, Z851, Z942, Z943**.** The population at risk of dialysis included the patients with chronic renal disease and without dialysis in the 1-year period preceding the index sepsis episode. Chronic renal disease was considered if one of the following ICD-10 codes was reported during at least one hospitalization or one hospital outpatient or ambulatory visits in the 1-year period preceding the index episode of sepsis: N03, N118, N119, N18, N19.

- **Long-term mechanical ventilation**

ICD-10 Codes: Z990, Z991

or

CCAM Codes: GLLD002, GLLD003, GLLP003, GLMP001, GLRP002

- **Dialysis**

ICD-10 Codes: Z49, Z992

or

CCAM Codes: JVJB001, JVJF003, JVJF004, JVJF008, JVRP004, JVRP007, JVRP008, YYYY007

Table S1 ICD-10 codes used to identify sepsis of presumed bacterial etiology according to type of selection in sepsis patients >15yrs^8^

| **Explicit sepsis codes*^a,b,d^*** | **Implicit sepsis *^b,c,d^*** | | |
| --- | --- | --- | --- |
|  | **Infection codes*^a^*** | **1^st^ associated condition** | **2^nd^ associated condition** |
| Sepsis of presumed bacterial etiology | |  |  |
| A021, A400-A409, A410-A419, A480, A483, O85, O883, R572, R578, R651 | A040-A049, A390-A399, G000-G009, I330, J068, J13, J14, J150-J159, J160-J168, J180-J189, J869, K650, K659, K810, K830, L022, L089, M000-M0099, M4620-M4629, M6000-M6009, M8600-M8609, M8690-M8699, N136, N390, T793, T802, T811, T814, T827, T845, T857 | ICU admission | ICD-10 codes for organ dysfunction: A483, D65, D689, D695, D696, D762, E86, E872, F050-F059, F09, G934, I460, I469, I950, I959, J80, J81, J952, J969, K720, K729, N080, N088, N160, N170-N179, N19, R092, R170-179, R34, R400-R4028, R392, R410, R418, R55, R571, R579  CCAM codes for organ support: DKMD001, DKMD002, EQLF002, EQLF003, FELF003, GLLD003, GLLD004, GLLD008, GLLD012, GLLD015, GLLD019, JVJB002, JVJF002, JVJF003, JVJF005, |

*^a^ One of the ICD-10 code as primary diagnosis (PD: condition requiring hospitalization), related diagnosis (RD: adds information to PD) or significant associated diagnosis (SAD: complications and co-morbidities potentially affecting the course or cost of hospitalization)*

*^b^ Sepsis = sepsis explicit sepsis + implicit sepsis*

*^c^ Implicit sepsis = ICD-10 code of infection + ICU admission + organ dysfunction/support*

*^d^ Stays shorter than 24h hours without death were excluded from our selection*

Table S2 Description of the variables

| **Variables** | **Sub-categories** |
| --- | --- |
| Sex | Male, Female |
| Age | 16-25, 26-35, 36-45, 46-55, 56-65, 66-75, 75-85, >85 |
| Charlson Index | 0, 1-2, 3-4, >4 based on the modified classification of Quan et al. (2011)^9^ |
| Comorbidities | Heart failure, Dementia, Chronic pulmonary diseases, Liver disease (mild, moderate or severe liver diseases), Diabetes with chronic complications, Paraplegia et hemiplegia, Renal disease, Cancer (cancer or metastatic carcinoma), AIDS/HIV. Based on the modified classification of Quan *et al.* (2011)^9 a^ |
| Hospital discharge | Acute care (To a short hospital stay in medicine, surgery or obstetrics units, after a transfer for or after a medical procedure or from psychiatry unit), Long-term care (To follow-up and rehabilitation care unit or long-term care unit or hospital-at-home), Home, Death. |
| Length of stay (days) | Number of days from date of admission to date of discharge, further stratified in 4 groups <7days, 7-14 days, 15-30 days, >30 days |
| ICU admission | ICU admission: recorded in one of the following medical unit: Intensive care unit (ICU), Other ICU, Coronary care unit, Neuro-intensive care; No ICU admission: not recorded in one of the above listed units |
| Septic shock | Septic shock: ICD-10 codes R572, R578 as primary diagnosis, related diagnosis or significant associated diagnosis, No septic shock: No ICD-10 codes R572, R578 as primary diagnosis, related diagnosis or significant associated diagnosis |
| Infection site | Abdomen and digestive tract, primary bacteremia, Bones and joints, Heart and mediastinum, Multiple sites, Associated with medical device, Lower respiratory tract, Skin and soft tissues, Urinary and genital tracts or other sites |

*^a^ cancer and metastatic carcinoma were classified as cancer; mild and severe liver disease were classified as liver disease.*

**Fig. S1** - Number of patients and percentage of deaths in the 3 consecutive years post-sepsis (2016-2018)

Table S3 Cognitive, psychological, and medical impairment for sepsis survivors in the 1-year period following the index sepsis episode

|  | Cluster 1: | | Cluster 2: | | Cluster 3: | | Cluster 4: | | Cluster 5: | | All survivors | |
| --- | --- | --- | --- | --- | --- | --- | --- | --- | --- | --- | --- | --- |
| Variables^a^ | Early  death | | Late  death | | Short-term  rehabilitation | | Long-term  rehabilitation | | Home | |  |  |
|  | N=19003 | | N=10058 | | N=16597 | | N=4865 | | N=96490 | | N=147013 | |
| Prevalence medical impairment | 18511 | 97.4 | 9860 | 98.0 | 16261 | 98.0 | 4763 | 97.9 | 82633 | 85.6 | 132028 | 89.8 |
| Incidence medical impairment | 16699 | 87.9 | 9173 | 91.2 | 15409 | 92.8 | 4613 | 94.8 | 70543 | 73.1 | 116434 | 79.2 |
| Prevalence psychological impairment | 6298 | 33.1 | 4144 | 41.2 | 8340 | 50.3 | 2818 | 57.9 | 32854 | 34.1 | 54454 | 37.0 |
| Incidence psychological impairment | 4294 | 22.6 | 2981 | 29.6 | 6535 | 39.4 | 2252 | 46.3 | 23523 | 24.4 | 39584 | 26.9 |
| Prevalence cognitive impairment | 6714 | 35.3 | 3774 | 37.5 | 6352 | 38.3 | 1937 | 39.8 | 18745 | 19.4 | 37522 | 25.5 |
| Incidence cognitive impairment | 4250 | 22.4 | 2585 | 25.7 | 5064 | 30.5 | 1650 | 33.9 | 13600 | 14.1 | 27142 | 18.5 |
| Prevalence of mechanical ventilation | 97 | 0.5 | 568 | 5.7 | 604 | 3.6 | 314 | 6.45 | 2335 | 2.4 | 3918 | 2.7 |
| Incidence of long-term mechanical ventilation (LTMV) | 83 | 0.4 | 448 | 4.5 | 495 | 3.0 | 270 | 5.6 | 1846 | 1.9 | 3142 | 2.1 |
| LTMV in patients at risk | 31 | 0.8 | 182 | 8.4 | 224 | 9.5 | 91 | 16.4 | 758 | 6.3 | 1286 | 6.1 |
| (Total number of patients at risk) | (4007) |  | (2162) |  | (2366) |  | (556) |  | (11979) |  | (21070) |  |
| Prevalence of dialysis | 902 | 4.8 | 601 | 6.0 | 716 | 4.3 | 291 | 6.0 | 4303 | 4.5 | 6816 | 4.6 |
| Incidence of dialysis | 418 | 2.2 | 307 | 3.1 | 520 | 3.1 | 189 | 3.9 | 2019 | 2.1 | 3453 | 2.4 |
| Incidence of dialysis in patients at risk | 157 | 7.3 | 129 | 11.4 | 159 | 12.3 | 56 | 20.3 | 824 | 13.7 | 1325 | 12.2 |
| (Total number of patients at risk) | (2163) |  | (1131) |  | (1295) |  | (276) |  | (6011) |  | (10876) |  |

*^a^ Data are reported as number of patients and percent in each group or cluster*

**Table S4 -** Proportion of patients with ambulatory care, for each cluster, in the 1-year period pre and post sepsis and number of visits

|  | **Cluster 1:** | | **Cluster 2:** | | **Cluster 3:** | | **Cluster 4:** | | | **Cluster 5:** | | | | **All survivors** | |
| --- | --- | --- | --- | --- | --- | --- | --- | --- | --- | --- | --- | --- | --- | --- | --- |
|  | **Early**  **death** | | **Late  death** | | **Long-term**  **rehabilitation** | | **Short-term**  **rehabilitation** | | | **Home** | | | |  |  |
| **Ambulatory care 1-year pre-sepsis** | | | | | | | | | | | | | | | |
| **Nursing care and physiotherapy ^a^** | | |  |  |  |  |  | |  |  | |  | |  |  |
| Patients, N, % | 15089 | 79.4 | 7893 | 78.5 | 12100 | 72.9 | 2828 | | 58.1 | 64149 | | 66.5 | | 102059 | 69.4 |
| Visits, median [IQR] ^b^ | 30 [7 - 116] | | 28 [6 - 109] | | 21 [4 - 93] | | 18 [3 - 89] | | | 13 [3 - 53] | | | | 16 [3 - 71] | |
| **GP visits** |  | |  | |  | |  | | |  | | | |  | |
| Patients, N, % | 18280 | 96.2 | 9681 | 96.3 | 15740 | 94.8 | 4354 | | 89.5 | 89882 | | 93.2 | | 137937 | 93.8 |
| Visits, median [IQR] ^b^ | 11 [6 - 16] | | 10 [6 - 16] | | 9 [5 - 14] | | 7 [4 - 12] | | | 8 [5 - 13] | | | | 9 [5 - 14] | |
| **Specialist visits ^c^** |  |  |  |  |  |  |  | |  |  | |  | |  |  |
| Patients, N, % | 11810 | 62.2 | 6429 | 63.9 | 10061 | 60.6 | 2510 | | 51.6 | 58304 | | 60.4 | | 89114 | 60.6 |
| Visits, median [IQR] ^b^ | 3 [2 - 8] | | 4 [2 - 8] | | 3 [2 - 7] | | 3 [1 - 7] | | | 3 [2 - 7] | | | | 3 [2 - 7] | |
| **Hospital outpatient visits** |  |  |  |  |  |  |  | |  |  | |  | |  |  |
| Patients, N, % | 15503 | 81.6 | 8360 | 83.1 | 12086 | 72.8 | 3465 | | 71.2 | 72867 | | 75.5 | | 112281 | 76.4 |
| Visits, median [IQR] ^d^ | 6 [3 - 12] | | 6 [3 - 12] | | 4 [2 - 13] | | 5 [2 - 10] | | | 5 [2 - 11] | | | | 5 [2 – 10] | |
| **Ambulatory care 1-year post-sepsis** | | | | | | | | | | | | | | | |
| **Nursing care and physiotherapy ^a^** | | |  |  |  |  |  |  | | |  | |  |  |  |
| Patients, N, % | 6013 | 31.6 | 6914 | 68.7 | 13032 | 78.5 | 2802 | 57.6 | | | 70208 | | 72.8 | 98969 | 67.3 |
| Visits, median [IQR] ^b^ | 11 [4 - 25] | | 33 [9 - 88] | | 52 [14 - 164] | | 31 [8 - 88] | | | | 23 [5 - 88] | | | 25 [6 - 89] | |
| **GP visits** |  |  |  |  |  |  |  |  | | |  | |  |  |  |
| Patients, N, % | 9548 | 50.2 | 9088 | 90.4 | 15697 | 94.6 | 4351 | 89.4 | | | 90105 | | 93.4 | 128789 | 87.6 |
| Visits, median [IQR] ^b^ | 3 [1 - 5] | | 7 [3 - 11] | | 9 [6 - 14] | | 5 [2 - 9] | | | | 9 [5 - 15] | | | 8 [4 - 14] | |
| **Specialist visits ^c^** |  |  |  |  |  |  |  |  | | |  | |  |  |  |
| Patients, N, % | 3412 | 18.0 | 4902 | 48.7 | 10912 | 65.8 | 2,651 | 54.5 | | | 64287 | | 66.6 | 86164 | 58.6 |
| Visits, median [IQR] ^b^ | 2 [1 - 7] | | 3 [1 - 6] | | 4 [2 - 7] | | 3 [1 - 6] | | | | 4 [2 - 7] | | | 4 [2 - 7] | |
| **Hospital outpatient visits** |  |  |  |  |  |  |  |  | | |  | |  |  |  |
| Patients, N, % | 7404 | 39.0 | 8164 | 81.2 | 14841 | 89.4 | 4390 | 90.2 | | | 81158 | | 84.1 | 115957 | 78.9 |
| Visits, median [IQR] ^d^ | 2 [1 - 5] | | 5 [2 - 10] | | 10 [4 - 22] | | 10 [4 - 39] | | | | 6 [3 - 12] | | | 6 [3 - 13] | |

^a^ Nurse, physiotherapist or speech therapist

^b^ Number of visits amongst patients who had at least one visit. A visit could include several visits or medical cares occurring on the same day; however, several visits could occur on the same day only if the visits concerned different medical or paramedical disciplines.

^c^ Cardiologist, dermatologist, gastroenterologist, internal medicine, respirologist, rheumatologist, physical and readaptation medicine, neurologist, psychiatrist, nephrologist, endocrinologist, surgeon

^d^ Number of hospital outpatient visits (only amongst patients who had at least one hospital outpatient care). A visit could include several visits or medical care occurring on the same day. The difference between medical subspecialties could not be made.

Table S5 Mean number of ambulatory visits and mean time spent hospitalized or at home, during the year pre- and post-sepsis, for each cluster

|  | Cluster 1 | Cluster 2 | Cluster 3 | Cluster 4 | Cluster 5 | |
| --- | --- | --- | --- | --- | --- | --- |
|  | Early  death | Late  death | Short-term  rehabilitation | Long-term  rehabilitation | Home | |
|  | N=19003 | N=10058 | N=16597 | N=4865 | N=96490 | |
| 1-year pre-sepsis | | | | | |  |
| Mean number of ambulatory visits |  |  |  |  |  | |
| Nursing care and physiotherapy ^a^ | 70.6 | 67.8 | 60.1 | 43.2 | 39.5 | |
| General Practitioner visits | 12.1 | 11.7 | 9.8 | 8.1 | 9.3 | |
| Specialist visits ^b^ | 5.3 | 5.3 | 4.0 | 4.6 | 4.2 | |
| Hospital outpatient visits ^c^ | 8.6 | 9.1 | 6.7 | 8.5 | 7.2 | |
| Mean number of days hospitalized in: |  |  |  |  |  | |
| Inpatient hospitalization in acute care | 21.5 | 21.0 | 12.7 | 14.6 | 12.0 | |
| Day care in acute care | 9.2 | 8.9 | 2.7 | 4.4 | 4.7 | |
| Inpatient hospitalization in rehabilitation | 8.3 | 7.2 | 9.2 | 15.3 | 3.3 | |
| Day care in rehabilitation | 0.5 | 0.5 | 0.8 | 1.2 | 0.5 | |
| Hospital-at-home | 3.3 | 2.3 | 0.5 | 1.9 | 1.1 | |
| Psychiatry | 1.1 | 1.7 | 1.7 | 3.3 | 2.0 | |
| 1-year post-sepsis | | | | | |  |
| Mean number of ambulatory visits |  |  |  |  |  | |
| Nursing care and physiotherapy ^a^ | 5.9 | 40.1 | 80.7 | 34.7 | 56.1 | |
| GP visits | 2.1 | 8.0 | 10.4 | 6.0 | 10.3 | |
| Specialist visits ^b^ | 1.3 | 4.4 | 5.3 | 5.0 | 5.4 | |
| Hospital outpatient visits ^c^ | 2.2 | 7.5 | 15.9 | 27.0 | 9.2 | |
| Mean number of days hospitalized in: |  |  |  |  |  | |
| Inpatient hospitalization in acute care | 14.9 | 38.0 | 21.8 | 33.6 | 16.1 | |
| Day care in acute care | 1.6 | 7.9 | 5.4 | 9.0 | 7.0 | |
| Inpatient hospitalization in rehabilitation | 10.3 | 18.3 | 59.4 | 194.7 | 4.9 | |
| Day care in rehabilitation | 0.1 | 0.3 | 6.1 | 18.5 | 1.3 | |
| Hospital-at-home | 4.3 | 10.8 | 3.3 | 9.1 | 3.6 | |
| Psychiatry | 0.1 | 0.8 | 1.7 | 3.0 | 2.2 | |
|  |  |  |  |  |  | |
| Mean number of days at home | 14.2 | 114.5 | 269.1 | 103.4 | 330.6 | |
|  |  |  |  |  |  | |
| Mean number of days alive | 43.1 | 186.6 | 364.1 | 361.4 | 364.6 | |
| Hospitalization and ambulatory visits ratios (1-year post sepsis) | | | | | |  |
| Mean % of days occupied by ambulatory visits in ^d^: |  |  |  |  |  | |
| Nursing care and physiotherapy ^a^ | 25.4% | 28.5% | 27.4% | 24.3% | 16.0% | |
| GP visits | 14.2% | 7.1% | 3.8% | 5.8% | 3.2% | |
| Specialist visits ^b^ | 5.1% | 3.9% | 2.0% | 4.7% | 1.8% | |
| Hospital outpatient visits ^c^ | 7.2% | 6.6% | 5.7% | 19.7% | 2.8% | |
| Mean number of days available for ambulatory visits | 18.6 | 129.8 | 281.6 | 131.4 | 341.9 | |
| Mean % of days occupied by hospitalization in ^e^: |  |  |  |  |  | |
| Inpatient hospitalization in acute care | 37.0% | 20.2% | 6.0% | 9.3% | 4.4% | |
| Day care in acute care | 2.9% | 3.9% | 1.5% | 2.5% | 1.9% | |
| Inpatient hospitalization in rehabilitation | 22.7% | 9.5% | 16.3% | 54.1% | 1.3% | |
| Day care in rehabilitation | 0.1% | 0.1% | 1.7% | 5.0% | 0.4% | |
| Hospital-at-home | 0.9% | 0.1% | 0.0% | 0.0% | 0.0% | |
| Psychiatry | 0.2% | 0.4% | 0.5% | 0.8% | 0.6% | |
| Mean % of days at home ^f^ | 32.4% | 62.8% | 73.9% | 28.4% | 90.6% | |
| Mean number of days available for hospitalization | 43.1 | 186.6 | 364.1 | 361.4 | 364.6 | |

*^a^ Nurse, physiotherapist or speech therapist visits were calculated separately and combined to calculate the final number of visits. Several visits to the same therapist in the same day were considered as a unique visit.*

*^b^ Cardiologist, dermatologist, gastroenterologist, internal medicine, respirologist, rheumatologist, physical and readaptation medicine, neurologist, psychiatrist, nephrologist, endocrinologist, surgeon visits were calculated separately and combined to calculate the final number of visits. Several visits to the same specialist in the same day were considered as a unique visit.*

*^c^ Several visits could occur in the same day*

*^d^ (Number of days with ambulatory visits during the following year/ Number of days available) x100. Days available=days where the patient is neither dead nor hospitalized*

*^e^ (Number of days of hospitalization during the following year/ Number of days available) x100. Days available=days where the patient is not dead*

*^f^ (Number of days at home during the following year/ Number of days available) x100. Days available=days where the patient is not dead*

**Fig. S2** - Percentage of surviving patients with hospitalizations and ambulatory visits amongst sepsis survivors in the 3 consecutive years following their index sepsis hospitalization.

Table S6 Mean and 95% confidence interval [95CI] of the cost in euros per patient for the index hospitalization and the 1-year period pre- and post-sepsis care

| Mean [95CI] cost per patients in € | Cluster 1:  Early  death | Cluster 2:  Late  death | Cluster 3:  Short-term  rehabilitation | Cluster 4:  Long-term  rehabilitation | Cluster 5:  Home | All survivors |  |
| --- | --- | --- | --- | --- | --- | --- | --- |
|  | N=19003 | N=10058 | N=16597 | N=4865 | N=96490 | N=147013 |  |
| Pre-sepsis | | | | | | | |
| Community ambulatory visits | 11199 [11004-11393] | 10724 [10467-10981] | 6982 [6837-7127] | 7844 [7482-8206] | 7235 [7126-7345] | 7992 [7911-8073] |  |
| Hospital outpatient visits | 1470 [1412-1529] | 1533 [1447-1619] | 1190 [1132-1248] | 1884 [1704-2065] | 1229 [1199-1258] | 1298 [1275-1321] |  |
|  |  |  |  |  |  |  |  |
| Inpatient or day care in acute care | 14899 [14664-15134] | 15053 [14702-15403] | 10321 [10037-10605] | 12359 [11764-12953] | 11174 [11046-11301] | 12074 [11974-12173] |  |
| Inpatient or day care in rehabilitation | 14040[13309-14770] | 13417[12376-14457] | 13554[12856-14251] | 27981[26455-29506] | 12452[12016-12887] | 15107[14757-15457] |  |
| Index hospitalization | | | | | | | |
|  | 12654 [12440-12868] | 12999 [12694-13304] | 21968 [21607-22328] | 33038 [32215-33861] | 12978 [12889-13066] | 14675 [14589-14762] |  |
| Post-sepsis | | | | | | | |
| Community ambulatory visits | 4018 [3911-4125] | 10683 [10414-10951] | 11349 [11165-11534] | 20340 [19647-21034] | 9056 [8919-9194] | 9293 [9191-9394] |  |
| Hospital outpatient visits | 467 [443-491] | 1427 [1354-1500] | 3540 [3453-3627] | 13758 [13151-14365] | 1302 [1273-1331] | 1867 [1836-1899] |  |
|  |  |  |  |  |  |  |  |
| Inpatient or day care in acute care | 11489 [11263-11715] | 22682 [22183-23181] | 14045 [13755-14335] | 19940 [19284-20597] | 14837 [14669-15004] | 15320 [15193-15447] |  |
| Inpatient or day care in rehabilitation | 12167 [11666-12669] | 17892 [17194-18589] | 19395 [18936-19854] | 61662 [59858-63466] | 11088 [10754-11422] | 21730 [21333-22126] |  |

Table S7 Total cost of sepsis and median and interquartile cost per patient for the index hospitalization and the 1-year period pre- and post-sepsis care

|  | | **CLUSTER 1:** | **CLUSTER 2:** | **CLUSTER 3:** | **CLUSTER 4:** | **CLUSTER 5:** | **All survivors** | |
| --- | --- | --- | --- | --- | --- | --- | --- | --- |
|  | **Early death** | | **Late death** | **Short-term**  **rehabilitation** | **Long-term**  **rehabilitation** | **Home** |  | |
| **1-year pre-sepsis** | | | | | | | |  |
|  | **€** | | **€** | **€** | **€** | **€** | **€** | |
| **Ambulatory cost** | 238912753 | | 122359471 | 132971135 | 45509655 | 793749978 | 1333502993 | |
| **Community ambulatory visits** |  | |  |  |  |  |  | |
| Cost per patient (median. IQR) | 5370 [2039 –13614] | | 5202 [1994 – 12951] | 3242 [1182 – 8716] | 2428 [555 – 9112] | 2812 [1028 – 7659] | 3240 [1172 – 8949] | |
| Total cost | 210971281 | | 106939608 | 113215748 | 36342424 | 675200580 | 1142669641 | |
| **Hospital outpatient visits** |  | |  |  |  |  |  | |
| Cost per patient (median. IQR) | 260 [0 –1024] | | 292 [0 – 1106] | 103 [0-575] | 80 [0-550] | 154 [0-656] | 165 [0-711] | |
| Total cost | 27941472 | | 15419864 | 19755386 | 9167231 | 118549399 | 190833352 | |
| **Inpatient and day care hospital cost^a^** | 237121205 | | 127504389 | 123155055 | 73720632 | 640464574 | 1201965855 | |
| **Acute care** |  | |  |  |  |  |  | |
| Cost per patient (median. IQR) | 9962 [4585 – 19850] | | 9502 [4325 – 19615] | 6132 [2847 – 12646] | 7471 [2839 – 15831] | 5727 [2460 – 13264] | 6696 [2881 – 15055] | |
| Total cost | 189037910 | | 103156828 | 84674026 | 27115082 | 527881144 | 931864990 | |
| Total extra medication costs ^b^ | 20107337 | | 10733002 | 5204418 | 1188683 | 50543341 | 87776781 | |
| **Rehabilitation** |  | |  |  |  |  |  | |
| Cost per patient (median. IQR) | 8265 [2880 – 17640] | | 7967 [2753 – 17168] | 7898 [2727 – 16787] | 14323 [2732 – 37397] | 6720 [2194 – 14932] | 7816 [2467 – 17906] | |
| Total cost | 27925227 | | 13564341 | 33179687 | 45272765 | 61885543 | 181827564 | |
| Total extra medication costs ^b^ | 50730 | | 50219 | 96924 | 144102 | 154545 | 496521 | |
| **Incident hospitalisation** | | | | | | | |  |
| Total cost | 206597729 | | 113284595 | 321671556 | 148968495 | 1085403643 | 1875926017 | |
| Cost per patients (median. IQR) | 8087 [5944 – 13273] | | 8006 [5670 – 14143] | 14846 [7685 – 27127] | 23754 [10776 –42688] | 8042 [4825 – 16411] | 8748 [5347 – 17867] | |
| Extra medication costs per patient^b^ | 0 [0-0] | | 0 [0-0] | 0 [0-0] | 0 [0-0] | 0 [0-0] | 0 [0-0] | |
| Total extra medication costs ^b^ | 4931126 | | 4510119 | 7198709 | 2882039 | 29963560 | 49485554 | |
| **1-year post-sepsis** | | | | | | | |  |
| **Ambulatory cost** | 68430813 | | 119910951 | 244369850 | 164443890 | 974501271 | 1571656775 | |
| **Community ambulatory visits** |  | |  |  |  |  |  | |
| Cost per patient (median. IQR) | 1098 [178 – 4586] | | 4722 [1542 – 12843] | 7168 [2624 – 14844] | 6349 [1194 – 30067] | 3863 [1466 – 10195] | 3912 [1314 – 10683] | |
| Total cost | 59561963 | | 105555795 | 185617851 | 97510987 | 848864579 | 1297111175 | |
| **Hospital outpatient visits** |  | |  |  |  |  |  | |
| Cost per patient (median. IQR) | 0 [0-94] | | 232 [46-676] | 525 [134 – 4654] | 503 [132 – 19618] | 283 [66-793] | 243 [26-802] | |
| Total cost | 8868850 | | 14355156 | 58751999 | 66932902 | 125636692 | 274545600 | |
| **Inpatient and day care hospital cost^a^** | 137414420 | | 230776399 | 258143806 | 235663153 | 957750976 | 1819748754 | |
| **Acute care** |  | |  |  |  |  |  | |
| Cost per patient (median. IQR) | 7555 [4430 – 14295] | | 15321 [7424 – 28030] | 8652 [3848 – 17574] | 12390 [5027 – 26161] | 7125 [2860 – 17063] | 8169 [3412 – 18347] | |
| Total cost | 116853980 | | 191053239 | 154045905 | 74616902 | 809241880 | 1345811905 | |
| Total extra medication costs ^b^ | 5666907 | | 15313432 | 7957181 | 3733206 | 79961246 | 112631972 | |
| **Rehabilitation** |  | |  |  |  |  |  | |
| Cost per patient (median. IQR) | 4956 [956-11223] | | 7697 [1960-18238] | 10555 [3909-20542] | 39794 [14508-71000] | 5518 [1760-10710] | 8127 [2459-20036] | |
| Total cost | 14725258 | | 24309952 | 95567006 | 156521746 | 68326578 | 359450540 | |
| Total extra medication costs ^b^ | 168275 | | 99776 | 573713 | 791300 | 221272 | 1854337 | |

^a^ Hospitalization in acute care and rehabilitation only. Hospital-at-home and hospitalization in psychiatry are excluded

^b^ Supplementary cost for expensive medications not included in the cost of the hospital stay. Median cost per patients was null for all clusters: 0 [0-0].

Table S8 Total and median [IQR] cost in euros per survivor of ambulatory care and hospitalization during the 2^nd^ and 3^rd^ year post-sepsis.

|  | Cluster 1:  Early  death | Cluster 2:  Late  death | Cluster 3:  Short-term  rehabilitation | Cluster 4:  Long-term  rehabilitation | Cluster 5:  Home | All survivors |
| --- | --- | --- | --- | --- | --- | --- |
| **2^nd^ year post-sepsis** | N=0 | N=0 | N=15901 | N=4529 | N=92975 | N=113405 |
| **Ambulatory care** |  |  |  |  |  |  |
| Cost of community ambulatory visits (median. IQR^a^) | - | - | 4274 [1517 – 11203] | 7719 [2085 – 18834] | 2757 [1002 – 8157] | 3034 [1074 – 9059] |
| Total cost of community ambulatory visits | - | - | 128391522 | 61764980 | 655917190 | 846073693 |
| Cost of hospital outpatient visits (median. IQR^a^) | - | - | 145 [0 - 548] | 274 [40 - 2102] | 114 [0 - 412] | 121 [0 - 443] |
| Total cost of hospital outpatient visits | - | - | 21355765 | 34302940 | 72610583 | 128269287 |
| **Inpatient or day care** |  |  |  |  |  |  |
| Cost of acute care per patient (median. IQR^a^) | - | - | 6055 [2713 – 13087] | 6286 [2240 – 14854] | 5974 [2394 – 14247] | 6005 [2437 – 14081] |
| Total cost of acute care | - | - | 80525997 | 29844909 | 480051065 | 590421971 |
| Total extra medication costs in acute care^b^ | - | - | 5575330 | 1731933 | 52338013 | 59645276 |
| Cost of rehabilitation per patient (median. IQR^a^) | - | - | 11264 [3564 – 26195] | 14211 [2165 – 43518] | 10690 [3741 – 23389] | 11044 [3470 – 25890] |
| Total cost of rehabilitation | - | - | 36071118 | 40844146 | 88667704 | 165582968 |
| Extra medication costs in rehabilitation per patient | - | - | 0 [0 - 0] | 0 [0 - 0] | 0 [0 - 0] | 0 [0 - 0] |
| Total extra medication costs in rehabilitation | - | - | 42834 | 119560 | 150104 | 312499 |
| **3rd year post sepsis** | N=0 | N=0 | N=13900 | N=3947 | N=83883 | N=101730 |
| **Ambulatory care** |  |  |  |  |  |  |
| Cost of community ambulatory visits (median. IQR^a^) | - | - | 3926 [1354 – 10273] | 7776 [2342 – 18867] | 2557 [928 – 7514] | 2803 [992 – 8351] |
| Total cost of community ambulatory visits | - | - | 102236 911 | 51712643 | 536748727 | 690698280 |
| Cost of hospital outpatient visits (median. IQR^a^) | - | - | 45 [0 - 304] | 73 [0 - 393] | 56 [0 - 336] | 56 [0 - 334] |
| Total cost of hospital outpatient visits | - | - | 13511915 | 8798963 | 79688345 | 101999223 |
| **Inpatient or day care** |  |  |  |  |  |  |
| Cost of acute care per patient (median. IQR^a^) | - | - | 5787 [2631 – 12293] | 5609 [2185 – 12801] | 5473 [2250 – 12938] | 5524 [2315 – 12811] |
| Total cost of acute care | - | - | 59603341 | 19926201 | 358192813 | 437722356 |
| Total extra medication costs in acute care^b^ | - | - | 3868221 | 1329531 | 39806415 | 45004167 |
| Cost of rehabilitation per patient (median. IQR^a^) | - | - | 13789 [5460 – 28248] | 9057 [912 – 30957] | 11587 [4840 – 23596] | 11886 [4433 – 25166] |
| Total cost of rehabilitation | - | - | 29556496 | 21383576 | 78247554 | 129187626 |
| Extra medication costs in rehabilitation per patient | - | - | 0 [0 - 0] | 0 [0 - 0] | 0 [0 - 0] | 0 [0 - 0] |
| Total extra medication costs in rehabilitation | - | - | 14981 | 78228 | 56192 | 149401 |

^a^ *Interquartile range. ^b^* *Median cost per patients was null for all clusters: 0 [0-0].*

Fig. S3 Visualization of all care sequences without clustering.

On the y-axis, the mean time (number of weeks, 0 to 52) spent in the different states.


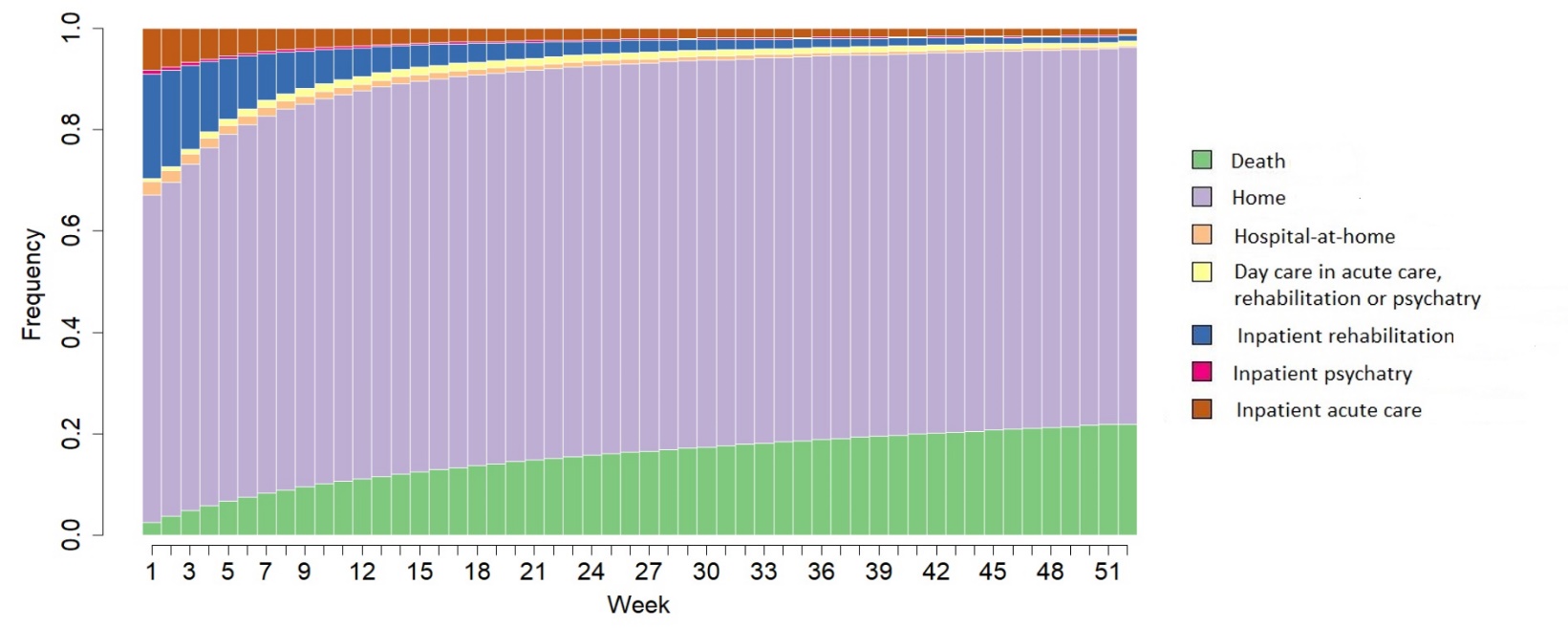


Fig. S4 Results of the Sequences and the clustering analyses

a. Plot of the sequence of modal states for each cluster (1 to 5**).** For each cluster (1 to 5), representation of the most frequent state for each weekly unit of the 1-year post sepsis care pathway. On the x-axis, time is graduated from discharge (week 1) to 1-year post-discharge (week 52). The y-axis corresponds to the proportion of patients (from 0 to 1). DEAD: death, MSO_full: Inpatient hospitalization in an acute care facility, REHAB_full: Inpatient hospitalization in a rehabilitation facility, PSY_full: Inpatient hospitalization in psychiatric facility, HAH: hospital-at-home, DAYCARE: day care in acute care, rehabilitation or psychiatric facility and HOME: home. On the y-axis, the mean time (number of weeks, 0 to 52) spent in the different states. Clusters determined by the state sequence analysis of the healthcare pathways of survivors: cluster 1 (early death), cluster 2 (late death), cluster 3 (short-term rehabilitation), cluster 4 (long-term rehabilitation), cluster 5 (home).

*
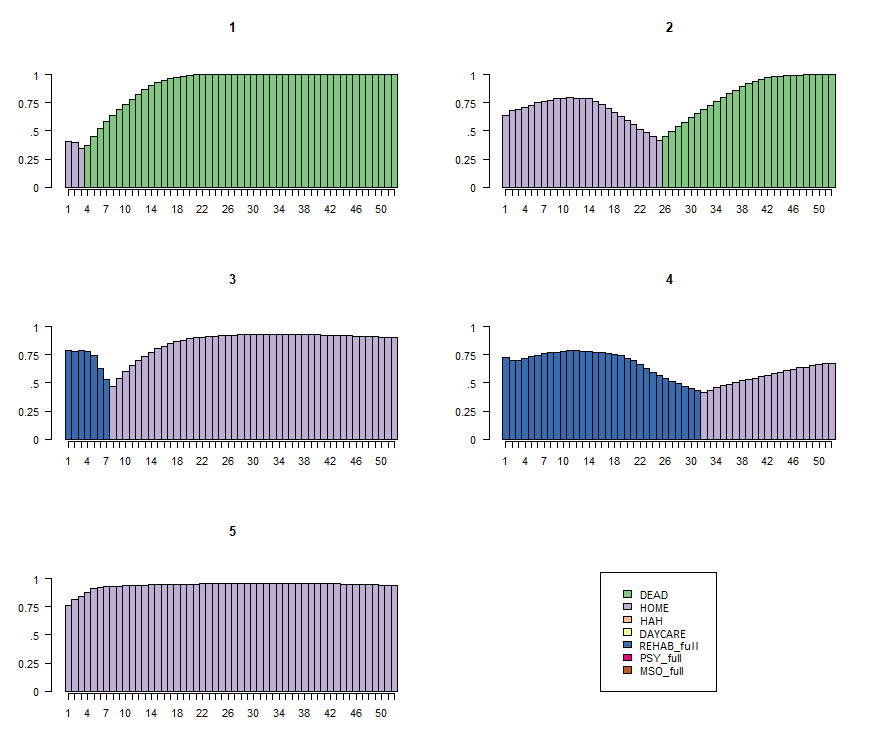
*

b. Plot of the mean time spent in each state for each cluster (1 to 5). For each cluster (1 to 5), mean time spent in each of the7 states**.** Clusters determined by the state sequence analysis of the healthcare pathways of survivors: cluster 1 (early death), cluster 2 (late death), cluster 3 (short-term rehabilitation), cluster 4 (long-term rehabilitation), cluster 5 (home).

*
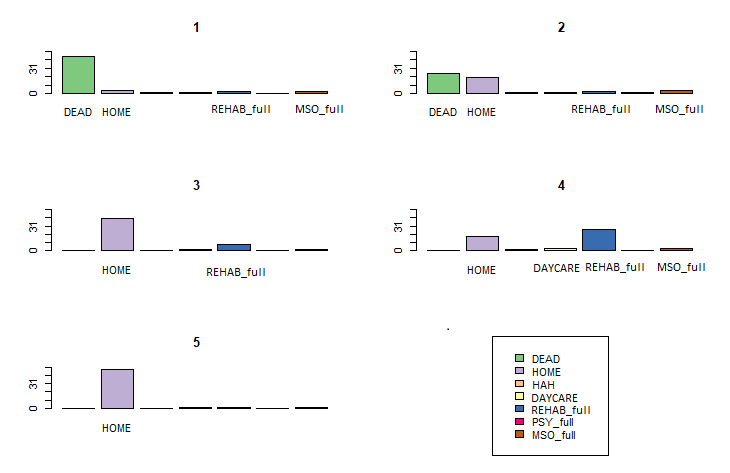
*

## c. Plot of transversal entropies for each Cluster (1 to 5)

Mean transversal entropy and plot of the transversal entropy along time. On the x-axis entropy index (0 to 1) for each weekly unit. The entropy is 0 when all patients are in the same state and the entropy is 1 when patients are equally distributed in each state. The entropy in the last weeks of the 1-year post-sepsis period in cluster 4 indicates lower homogeneity in the states of the patients compare to other clusters. Clusters determined by the state sequence analysis of the healthcare pathways of survivors: cluster 1 (early death), cluster 2 (late death), cluster 3 (short-term rehabilitation), cluster 4 (long-term rehabilitation), cluster 5 (home).

*
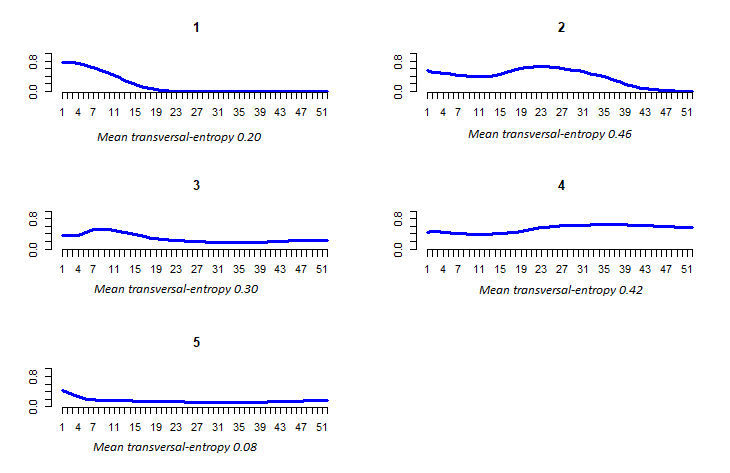
*

## d. Index plot of each cluster (1 to 5): individual sequences with stacked bars depicting the states over time

Each weekly states of all sequences stacked in one chronogramme for each cluster. States sequences tend to become homogeous in the last week of the 1_year period post-sepsis, except for cluster 4. Clusters determined by the state sequence analysis of the healthcare pathways of survivors: cluster 1 (early death), cluster 2 (late death), cluster 3 (short-term rehabilitation), cluster 4 (long-term rehabilitation), cluster 5 (home).

*
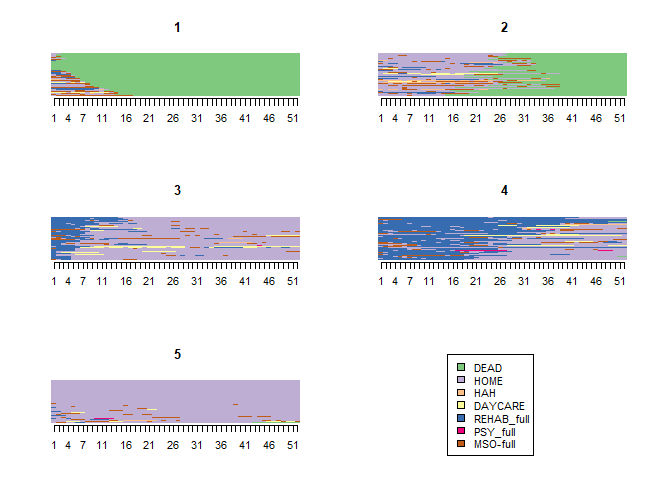
*

**Fig. S5** - Distribution of the infection sites across all survivors and in each cluster. Clusters determined by the state sequence analysis of the healthcare pathways of survivors: cluster 1 (early death), cluster 2 (late death), cluster 3 (short-term rehabilitation), cluster 4 (long-term rehabilitation), cluster 5 (home).

Table S9 Long-term assessment of hospitalization, mortality and ambulatory visits (2015-2018)

| **YEAR 1** | | | | | | | | | | | | |
| --- | --- | --- | --- | --- | --- | --- | --- | --- | --- | --- | --- | --- |
|  | **Cluster 1**  **Early death** | | **Cluster 2**  **Late death** | | **Cluster 3**  **Short-term rehabilitation** | | **Cluster 4**  **Long-term rehabilitation** | | **Cluster 5**  **Home** | | **All survivors** | |
|  | **N=19009** | | **N=10058** | | **N=16597** | | **N=4865** | | **N=96490** | | **N=147013** | |
| **Inpatient hospitalization in acute care** |  |  |  |  |  |  |  |  |  |  |  |  |
| Patients, N, % | 13175 | 69.3 | 9042 | 89.9 | 11904 | 71.7 | 3881 | 79.8 | 58059 | 60.2 | 96061 | 65.3 |
| Days, median [IQR^a^] | 16 [0 - 29] | | 32 [9 - 57] | | 21 [0 - 40] | | 28 [3 - 57] | | 14 [0 - 31] | | 17 [0 - 35] | |
| **Day care in acute care** |  |  |  |  |  |  |  |  |  |  |  |  |
| Patients, N, % | 4030 | 21.2 | 5030 | 50.0 | 5544 | 33.4 | 1822 | 37.5 | 36522 | 37.9 | 52948 | 36.0 |
| Days, median [IQR^a^] | 2 [1 - 5] | | 5 [1 - 12] | | 2 [1 - 7] | | 2 [1 - 7] | | 2 [1 - 10] | | 2 [1 - 9] | |
| **Inpatient hospitalization in rehabilitation** |  |  |  |  |  |  |  |  |  |  |  |  |
| Patients, N, % | 6355 | 33.4 | 3644 | 36.2 | 16597 | 100.0 | 4865 | 100.0 | 18227 | 18.9 | 49688 | 33.8 |
| Days, median [IQR^a^] | 24 [11 - 43] | | 40 [22 - 70] | | 53 [37 - 76] | | 177 [138 - 243] | | 22 [16 - 29] | | 35 [21 - 68] | |
| **Day care in rehabilitation** |  |  |  |  |  |  |  |  |  |  |  |  |
| Patients, N, % | 34 | 0.2 | 73 | 0.7 | 1502 | 9.1 | 1033 | 21.2 | 2596 | 2.7 | 5238 | 3.6 |
| Days, median [IQR^a^] | 19 [8 - 34] | | 32 [2 - 72] | | 52 [26 - 88] | | 73 [30 - 136] | | 33 [16 - 60] | | 43 [19 - 81] | |
| **Hospital-at-home** |  |  |  |  |  |  |  |  |  |  |  |  |
| Patients, N, % | 2855 | 15.0 | 1732 | 17.2 | 837 | 5.0 | 459 | 9.4 | 5473 | 5.7 | 11356 | 7.7 |
| Days, median [IQR^a^] | 19 [8 - 40] | | 35 [12 - 97] | | 41 [18 - 79] | | 73 [32 - 145] | | 32 [14 - 77] | | 29 [12 - 69] | |
| **Psychiatry** |  |  |  |  |  |  |  |  |  |  |  |  |
| Patients, N, % | 81 | 0.4 | 120 |  | 390 | 2.4 | 146 | 3.0 | 2431 | 2.5 | 3168 | 2.2 |
| Days, median [IQR^a^] | 19 [8 - 47] | | 33 [13 - 113] | | 42 [21 - 88] | | 58 [21 - 171] | | 41 [17 - 112] | | 40 [17 - 108] | |
| **Yearly mortality** | 19009 | 100 | 10058 | 100 | 696 | 4.2 | 336 | 6.91 | 3515 | 3.6 | 33608 | 22.9 |
| **Cumulative mortality** | 19009 | 100 | 10058 | 100 | 696 | 4.2 | 336 | 6.91 | 3515 | 3.6 | 33608 | 22.9 |
| **Nursing and physiotherapy** |  |  |  |  |  |  |  |  |  |  |  |  |
| Patients, N, % | 6013 | 31.6 | 6914 | 68.7 | 13032 | 78.5 | 2802 | 57.6 | 70208 | 72.8 | 98969 | 67.3 |
| Visits, median [IQR^a^] | 11 [4 - 25] | | 33 [9 - 88] | | 52 [14 - 164] | | 31 [8 - 88] | | 23 [5 - 88] | | 25 [6 - 89] | |
| **General practitioner visits** |  |  |  |  |  |  |  |  |  |  |  |  |
| Patients, N, % | 9548 | 50.2 | 9088 | 90.4 | 15697 | 94.6 | 4351 | 89.4 | 90105 | 93.4 | 128789 | 87.6 |
| Visits, median [IQR^a^] | 3 [1 - 5] | | 7 [3 - 11] | | 9 [6 - 14] | | 5 [2 - 9] | | 9 [5 - 15] | | 8 [4 - 14] | |
| **Specialist visits** |  |  |  |  |  |  |  |  |  |  |  |  |
| Patients, N, % | 3412 | 18.0 | 4902 | 48.7 | 10912 | 65.8 | 2651 | 54.5 | 64287 | 66.6 | 86164 | 58.6 |
| Visits, median [IQR^a^] | 2 [1 - 7] | | 3 [1 - 6] | | 4 [2 - 7] | | 3 [1 - 6] | | 4 [2 - 7] | | 4 [2 - 7] | |
| **Hospital outpatient visits** |  |  |  |  |  |  |  |  |  |  |  |  |
| Patients, N, % | 7404 | 39.0 | 8164 | 81.2 | 14841 | 89.4 | 4390 | 90.2 | 81158 | 84.1 | 115957 | 78.9 |
| Visits, median [IQR^a^] | 2 [1 - 5] | | 5 [2 - 10] | | 10 [4 - 22] | | 10 [4 - 39] | | 6 [3 - 12] | | 6 [3 - 13] | |

| **YEAR 2^b^** | | | | | | | | | |
| --- | --- | --- | --- | --- | --- | --- | --- | --- | --- |
|  | **Cluster 3**  **Short-term rehabilitation** | | **Cluster 4**  **Long-term rehabilitation** | | **Cluster 5**  **Home** | | | **All survivors** | |
|  | **N=15901** | | **N=4529** | | **N=92975** | | | **N=113405** | |
| **Inpatient hospitalization in acute care** |  |  |  |  |  |  |  | |  |
| Patients, N, % | 7482 | 47.1 | 2350 | 51.9 | 38,676 | 41.6 | 48,508 | | 42.8 |
| Days, median [IQR^a^] | 14 [6 - 29] | | 14 [6 - 30] | | 12 [5 - 28] | | 13 [6 - 28] | | |
| **Day care in acute care** |  |  |  |  |  |  |  | |  |
| Patients, N, % | 4051 | 25.5 | 1370 | 30.3 | 27,061 | 29.1 | 32,482 | | 28.6 |
| Days, median [IQR^a^] | 2 [1 - 5] | | 2 [1 - 5] | | 2 [1 - 7] | | 2 [1 - 6] | | |
| **Inpatient hospitalization in rehabilitation** |  |  |  |  |  |  |  | |  |
| Patients, N, % | 2536 | 16.0 | 1461 | 32.3 | 6,562 | 7.1 | 10,559 | | 9.3 |
| Days, median [IQR^a^] | 36 [21 - 63] | | 62 [27 - 145] | | 30 [18 - 54] | | 33 [20 - 63] | | |
| **Day care in rehabilitation** |  |  |  |  |  |  |  | |  |
| Patients, N, % | 517 | 3.3 | 813 | 18.0 | 1,214 | 1.3 | 2,544 | | 2.2 |
| Days, median [IQR^a^] | 28 [3 - 76] | | 55 [3 - 138] | | 29 [3 - 66] | | 33 [3 - 87] | | |
| **Hospital-at-home** |  |  |  |  |  |  |  | |  |
| Patients, N, % | 437 | 2.8 | 247 | 5.5 | 2,336 | 2.5 | 3,020 | | 2.7 |
| Days, median [IQR^a^] | 33 [13 - 83] | | 84 [28 - 179] | | 31 [12 - 80] | | 33 [13 - 88] | | |
| **Psychiatry** |  |  |  |  |  |  |  | |  |
| Patients, N, % | 271 | 1.7 | 114 | 2.5 | 1,588 | 1.7 | 1,973 | | 1.7 |
| Days, median [IQR^a^] | 45 [20 - 135] | | 57 [22 - 210] | | 44 [17 - 129] | | 44 [17 - 133] | | |
| **Yearly mortality** | 2001 | 12.6 | 582 | 12.9 | 9,092 | 9.8 | 11,675 | | 10.3 |
| **Cumulative mortality** | 2697 | 16.2 | 918 | 18.9 | 12,607 | 13.1 | 45,283 | | 30.80 |
| **Nursing and physiotherapy** |  |  |  |  |  |  |  | |  |
| Patients, N, % | 11453 | 72.0 | 3067 | 67.7 | 60,734 | 65.3 | 75,254 | | 66.4 |
| Visits, median [IQR^a^] | 49 [9 - 184] | | 76 [19 - 214] | | 16 [3 - 77] | | 20 [4 - 96] | | |
| **General practitioner visits** |  |  |  |  |  |  |  | |  |
| Patients, N, % | 14078 | 88.5 | 3941 | 87.0 | 82,472 | 88.7 | 100,491 | | 88.6 |
| Visits, median [IQR^a^] | 8 [5 - 14] | | 8 [4 - 13] | | 8 [4 - 12] | | 8 [4 - 13] | | |
| **Specialist visits** |  |  |  |  |  |  |  | |  |
| Patients, N, % | 9521 | 59.9 | 2571 | 56.8 | 57,205 | 61.5 | 69,297 | | 61.1 |
| Visits, median [IQR^a^] | 3 [2 - 6] | | 3 [1 - 6] | | 3 [2 - 6] | | 3 [2 - 6] | | |
| **Hospital outpatient visits** |  |  |  |  |  |  |  | |  |
| Patients, N, % | 11046 | 69.5 | 3566 | 78.7 | 63,703 | 68.5 | 78,315 | | 69.1 |
| Visits, median IQR^a^] | 4 [2 - 8] | | 5 [2 - 11] | | 4 [2 - 8] | | 4 [2 - 8] | | |

| **YEAR 3^b^** | | | | | | | | |
| --- | --- | --- | --- | --- | --- | --- | --- | --- |
|  | **Cluster 3**  **Short-term rehabilitation** | | **Cluster 4**  **Long-term rehabilitation** | | **Cluster 5**  **Home** | | **All survivors** | |
|  | **N=13900** | | **N=3947** | | **N=83883** | | **N=101730** | |
| **Inpatient hospitalization in acute care** |  |  |  |  |  |  |  |  |
| Patients, N, % | 5700 | 41.0 | 1694 | 42.9 | 30,615 | 36.5 | 38,009 | 37.4 |
| Days, median [IQR^a^] | 13 [6 - 28] | | 12 [5 - 27] | | 11 [5 - 25] | | 12 [5 - 26] | |
| **Day care in acute care** |  |  |  |  |  |  |  |  |
| Patients, N, % | 3131 | 22.5 | 1031 | 26.1 | 21,872 | 26.1 | 26,034 | 25.6 |
| Days, median [IQR^a^] | 1 [1 - 4] | | 2 [1 - 4] | | 2 [1 - 5] | | 2 [1 - 5] | |
| **Inpatient hospitalization in rehabilitation** |  |  |  |  |  |  |  |  |
| Patients, N, % | 1894 | 13.6 | 767 | 19.4 | 5,547 | 6.6 | 8,208 | 8.1 |
| Days, median [IQR^a^] | 37 [22 - 63] | | 50 [24 - 110] | | 30 [19 - 53] | | 32 [20 - 60] | |
| **Day care in rehabilitation** |  |  |  |  |  |  |  |  |
| Patients, N, % | 346 | 2.5 | 497 | 12.6 | 947 | 1.1 | 1,790 | 1.8 |
| Days, median [IQR^a^] | 26 [2 - 61] | | 23 [2 - 79] | | 28 [3 - 65] | | 26 [2 - 67] | |
| **Hospital-at-home** |  |  |  |  |  |  |  |  |
| Patients, N, % | 258 | 1.9 | 131 | 3.3 | 1,520 | 1.8 | 1,909 | 1.9 |
| Days, median [IQR^a^] | 32 [12 - 73] | | 64 [31 - 165] | | 31 [12 - 84] | | 33 [12 - 89] | |
| **Psychiatry** |  |  |  |  |  |  |  |  |
| Patients, N, % | 209 | 1.5 | 73 | 1.9 | 1,297 | 1.6 | 1,579 | 1.6 |
| Days, median [IQR^a^] | 41 [19 - 109] | | 47 [21 - 220] | | 44 [18 - 124] | | 44 [18 - 124] | |
| **Yearly mortality** | 1451 | 10.4 | 373 | 9.5 | 6,714 | 8.0 | 8,538 | 8.4 |
| **Cumulative mortality** | 4148 | 25.0 | 1291 | 26.5 | 19,321 | 20.0 | 53,821 | 36.6 |
| **Nursing and physiotherapy** |  |  |  |  |  |  |  |  |
| Patients, N, % | 7206 | 51.8 | 2092 | 53.0 | 37,201 | 44.4 | 46,499 | 45.7 |
| Visits, median [IQR^a^] | 23 [5 - 90] | | 41 [11 - 105] | | 9 [2 - 43] | | 12 [2 - 52] | |
| **General practitioner visits** |  |  |  |  |  |  |  |  |
| Patients, N, % | 11623 | 83.6 | 3222 | 81.6 | 70,666 | 84.2 | 85,511 | 84.1 |
| Visits, median [IQR^a^] | 7 [4 - 12] | | 7 [4 - 12] | | 7 [4 - 11] | | 7 [4 - 12] | |
| **Specialist visits** |  |  |  |  |  |  |  |  |
| Patients, N, % | 7946 | 57.2 | 2193 | 55.6 | 49,678 | 59.2 | 59,817 | 58.8 |
| Visits, median [IQR^a^] | 3 [2 - 6] | | 3 [2 - 6] | | 3 [2 - 6] | | 3 [2 - 6] | |
| **Hospital outpatient visits** |  |  |  |  |  |  |  |  |
| Patients, N, % | 8951 | 64.4 | 2853 | 72.3 | 63,703 | 68.5 | 65,955 | 64.8 |
| Visits, median [IQR^a^] | 3 [2 - 7] | | 4 [2 - 9] | | 4 [2 - 7] | | 4 [2 - 8] | |

*^a^ Interquartile range*

*^b^ Only patients still alive at the start of the year of interest are included in the table*


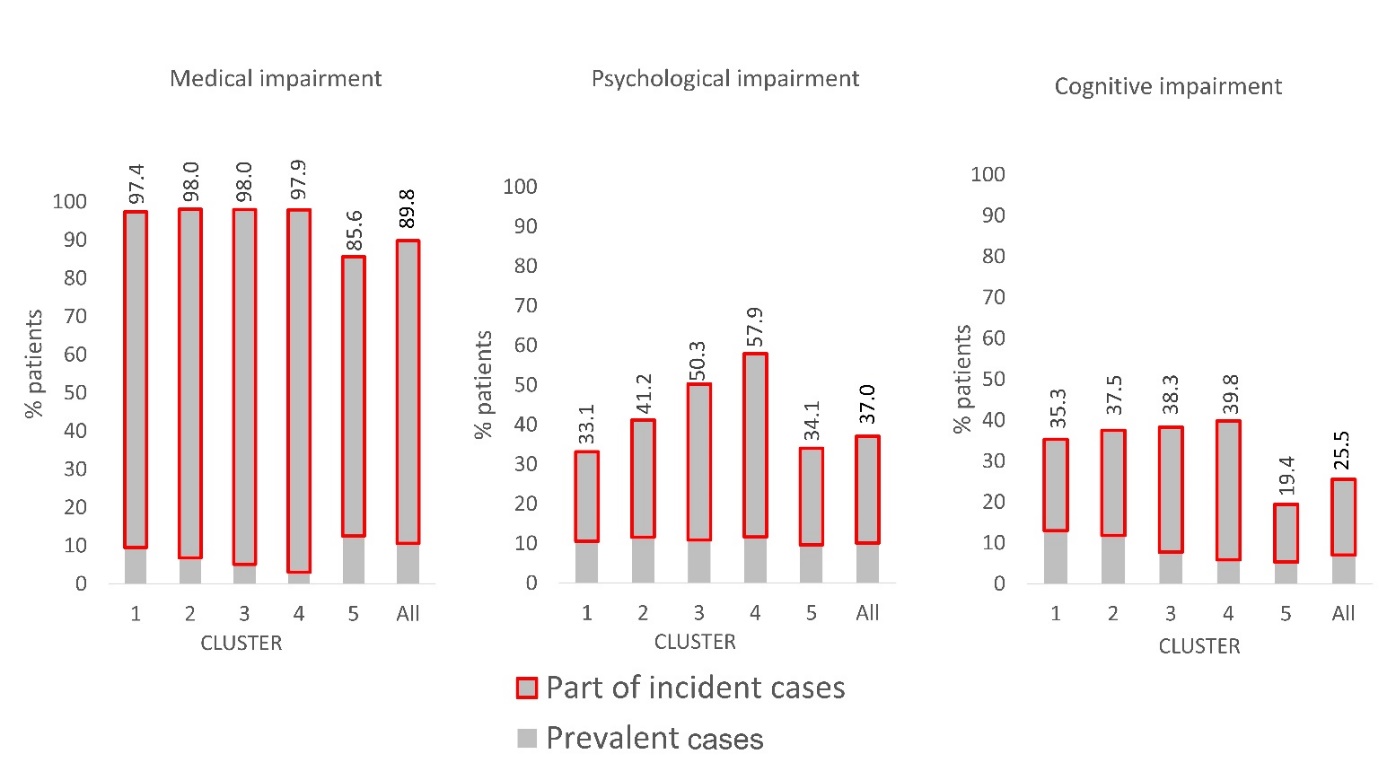
**Fig. S6** - Prevalent (in grey in the figure) and incident (in red on the figure) medical, psychological and cognitive impairments in sepsis survivors in the 1-year period following the index sepsis episode. Clusters determined by the state sequence analysis of the healthcare pathways of survivors: cluster 1 (early death), cluster 2 (late death), cluster 3 (short-term rehabilitation), cluster 4 (long-term rehabilitation), cluster 5 (home).

**References**

1. Tuppin P, Rudant J, Constantinou P, et al. Value of a national administrative database to guide public decisions: From the système national d’information interrégimes de l’Assurance Maladie (SNIIRAM) to the système national des données de santé (SNDS) in France. *Rev Epidemiol Sante Publique*. 2017;65 Suppl 4:S149-S167. doi:10.1016/j.respe.2017.05.004

2. Singer M, Deutschman CS, Seymour CW, et al. The Third International Consensus Definitions for Sepsis and Septic Shock (Sepsis-3). *JAMA*. 2016;315(8):801-810. doi:10.1001/jama.2016.0287

3. Gabadinho A, Ritschard G, Müller NS, Studer M. Analyzing and Visualizing State Sequences in R with TraMineR. *J Stat Softw*. 2011;40:1-37. doi:10.18637/jss.v040.i04

4. Roux J, Grimaud O, Leray E. Use of state sequence analysis for care pathway analysis: The example of multiple sclerosis. *Stat Methods Med Res*. 2019;28(6):1651-1663. doi:10.1177/0962280218772068

5. Touat M, Brun-Buisson C, Opatowski M, et al. Costs and Outcomes of 1-year post-discharge care trajectories of patients admitted with infection due to antibiotic-resistant bacteria. *J Infect*. 2021;82(3):339-345. doi:10.1016/j.jinf.2021.02.001

6. Studer M. WeightedCluster Library Manual: A practical guide to creating typologies of trajectories in the social sciences with R. *LIVES Work Pap*. 2013;24.

7. Fleischmann-Struzek C, Rose N, Freytag A, et al. Epidemiology and Costs of Postsepsis Morbidity, Nursing Care Dependency, and Mortality in Germany, 2013 to 2017. *JAMA Netw Open*. 2021;4(11):e2134290. doi:10.1001/jamanetworkopen.2021.34290

8. Pandolfi F, Brun-Buisson C, Guillemot D, Watier L. One-year hospital readmission for recurrent sepsis: associated risk factors and impact on 1-year mortality-a French nationwide study. *Crit Care Lond Engl*. 2022;26(1):371. doi:10.1186/s13054-022-04212-9

9. Quan H, Li B, Couris CM, et al. Updating and validating the Charlson comorbidity index and score for risk adjustment in hospital discharge abstracts using data from 6 countries. *Am J Epidemiol*. 2011;173(6):676-682. doi:10.1093/aje/kwq433
